# Vitamin D₃ Supplementation in Batswana Children and Adults with HIV: A Pilot Double Blind Randomized Controlled Trial

**DOI:** 10.1371/journal.pone.0117123

**Published:** 2015-02-23

**Authors:** Andrew P. Steenhoff, Joan I. Schall, Julia Samuel, Boitshepo Seme, Marape Marape, Bakgaki Ratshaa, Irene Goercke, Michael Tolle, Maria S. Nnyepi, Loeto Mazhani, Babette S. Zemel, Richard M. Rutstein, Virginia A. Stallings

**Affiliations:** 1 Botswana-UPenn Partnership, Gaborone, Botswana; 2 Division of Infectious Diseases, Children’s Hospital of Philadelphia, Philadelphia, Pennsylvania, United States of America; 3 Division of Gastroenterology, Hepatology and Nutrition, Children’s Hospital of Philadelphia, Philadelphia, Pennsylvania, United States of America; 4 Division of General Pediatrics, Children’s Hospital of Philadelphia, Philadelphia, Pennsylvania, United States of America; 5 Department of Pediatrics, Perelman School of Medicine at the University of Pennsylvania, Philadelphia, Pennsylvania, United States of America; 6 Botswana-Baylor Children’s Clinical Centre of Excellence, Gaborone, Botswana; 7 Department of Nutrition, University of Botswana, Gaborone, Botswana; 8 Department of Pediatrics and Adolescent Health, School of Medicine, University of Botswana, Gaborone, Botswana; The George Washington University Medical Center, UNITED STATES

## Abstract

**Objectives:**

Since vitamin D insufficiency is common worldwide in people with HIV, we explored safety and efficacy of high dose cholecalciferol (D₃) in Botswana, and evaluated potential modifiers of serum 25 hydroxy vitamin D change (Δ25D).

**Design:**

Prospective randomized double-blind 12-week pilot trial of subjects ages 5.0–50.9 years.

**Methods:**

Sixty subjects randomized within five age groups to either 4000 or 7000IU per day of D₃ and evaluated for vitamin D, parathyroid hormone, HIV, safety and growth status. Efficacy was defined as serum 25 hydroxy vitamin D (25D) ≥32ng/mL, and safety as no simultaneous elevation of serum calcium and 25D. Also assessed were HIV plasma viral RNA viral load (VL), CD4%, anti-retroviral therapy (ART) regime, and height-adjusted (HAZ), weight-adjusted (WAZ) and Body Mass Index (BMIZ) Z scores.

**Results:**

Subjects were 50% male, age (mean±SD) 19.5±11.8 years, CD4% 31.8±10.4, with baseline VL log₁₀ range of <1.4 to 3.8 and VL detectable (>1.4) in 22%. From baseline to 12 weeks, 25D increased from 36±9ng/ml to 56±18ng/ml (p<0.0001) and 68% and 90% had 25D ≥32ng/ml, respectively (p = 0.02). Δ25D was similar by dose. No subjects had simultaneously increased serum calcium and 25D. WAZ and BMIZ improved by 12 weeks (p<0.04). HAZ and CD4% increased and VL decreased in the 7000IU/d group (p<0.04). Younger (5–13y) and older (30–50y) subjects had greater Δ25D than those 14–29y (26±17 and 28±12 vs. 11±11ng/ml, respectively, p≤0.001). Δ25D was higher with efavirenz or nevirapine compared to protease inhibitor based treatment (22±12, 27±17, vs. 13±10, respectively, p≤0.03).

**Conclusions:**

In a pilot study in Botswana, 12-week high dose D₃ supplementation was safe and improved vitamin D, growth and HIV status; age and ART regimen were significant effect modifiers.

**Trial Registration:**

ClinicalTrials.gov NCT02189902

## Introduction

Suboptimal vitamin D status is common in people with HIV [1,2,3]. Inadequate sunlight, low dietary intake, increased utilization, drug therapies, malabsorption, or unknown HIV-associated factors may contribute [1,2,4,5]. Observational studies suggest that vitamin D status may impact HIV disease severity [1,6,7,8,9]. Serum 25-hydroxy vitamin D (25D) concentration was positively correlated with CD4^+^ counts in Norway [6], Germany [7,8] and in the USA [1]. Vitamin D supplementation was associated with changes in CD4 T-cell phenotype in Italy [9].

In Africa, vitamin D insufficiency and rickets are prevalent despite adequate sunlight exposure [10]. Insufficient 25D status (<32ng/ml) in Gambia was noted in 23% children and 55% of young women [11]. Data on vitamin D status in HIV-infected people from sub-Saharan Africa are limited. Among HIV-infected pregnant women in Tanzania, 39% had low 25D (<32ng/ml) with more rapid HIV progression, higher all-cause mortality and a higher incidence of anemia than women with higher 25D [12]. In HIV-infected adults from Botswana a quarter of subjects were vitamin D insufficient [13]. Despite these data suggesting that vitamin D insufficiency is prevalent in parts of Africa, the cholecalciferol (D_3_) dose needed to improve vitamin D status in this setting is unknown.

The objective of this pilot study was to test the safety and efficacy of two oral daily doses of D_3_ over 12 weeks in children and adults with HIV in Botswana.

## Patients and Methods

The protocol for this trial and supporting CONSORT checklist are available as supporting information; see S1 CONSORT Checklist and S1–S4 Protocols. Patients were recruited from 13^th^ December 2011 to 18^th^ April, 2012 from an outpatient clinic at Princess Marina Hospital in Gaborone, Botswana. The last 12 week follow-up visit was completed on 12^th^ July 2012. Eligible participants were aged 5.0 to 50.9 years, HIV infected, on first line antiretroviral therapy (ART), and in usual state of good health. “In usual state of good health” was defined as “no visit to a doctor, nurse or clinic for an acute medical condition over the 2 weeks prior to enrollment.” Exclusion criteria included HIV-unrelated chronic conditions that may affect growth, dietary intake or nutritional status. These chronic conditions were diarrhea for more than 14 days, malabsorption, renal disease requiring vitamin D supplementation, renal disease with a creatinine greater than 2.0mg/dL or oral steroid use for more than 14 days.

Sixty subjects were enrolled, 12 in each age group (5.0–8.9, 9.0–13.9, 14.0–18.9, 19.0–30.9, 31.0–50.9 years), and randomized to either 4000 or 7000IU/d of D_3_ for 12 weeks. Clinical data were extracted from medical records and a structured questionnaire. HIV was classified using the Centers for Disease Control and Prevention (CDC) clinical classification [14,15] and CD4 count.

ART regimens were characterized as tenofovir-containing, and either protease inhibitor-based (PI), or non-nucleoside reverse transcriptase inhibitor-based (NNRTI), being efavirenz or nevirapine. All subjects receiving tenofovir were also on NNRTIs.

Research nurses were trained in research quality anthropometric measurement (by JIS, APS). Height was measured by stadiometer (Seca, UK) and weight by digital standing scale (Adam Medical) for adults and a wheel chair digital scale (Seca, UK) in children. Body mass index (BMI) was calculated. Scales were calibrated weekly. Measurements were done in triplicate [16] and the mean used for analysis. For subjects under 20 years, weight, height and BMI were compared to reference standards to generate age- and sex-specific Z scores [17].

### Laboratory investigations

Tests performed at baseline, 6 and 12 weeks included: serum 25D using liquid chromatography tandem mass spectrometry (Clinical Laboratory, the Children’s Hospital of Philadelphia [CHOP], Philadelphia, PA, USA) with intra- and inter-assay coefficients of variation (CV) below 8%; serum 1,25-dihydroxyvitamin D (1,25D) and intact parathyroid hormone (PTH) by radioimmunoassay using a radio-iodinated tracer (Heartland Assays, Ames, IA, USA) with intra- and inter-assay CV of 9.8% and 12.6% for 1,25D and 2.7% and 4.3% for PTH; vitamin D binding protein (DBP) by enzyme linked immunosorbent assay (R&D Systems, Minneapolis, MN) with intra- and inter-assay CVs below 10%. Bioavailable 25D (ng/mL) was calculated as that unbound to DBP or albumin, and bioavailable/total 25(OH)D (%) derived [18]. Safety was assessed by serum albumin, calcium (corrected for albumin), magnesium, phosphorus and whole blood lead (Diagnofirm Diagnostic Laboratory, Gaborone, Botswana) using standard techniques. CD4 count, and HIV-1 RNA viral load (VL) were measured at baseline and 12 weeks at either Botswana Harvard or Diagnofirm Laboratories. Undetectable VL was defined as <25 copies/mL (RNA log ≤1.4). Per protocol analysis required assessment of serum cathelicidin antimicrobial protein activity—however the volume of blood collected from all subjects was insufficient to perform all per protocol tests. Hence the study team elected not to perform this test.

### Treatment and adherence

Study medication consisted of 4000IU/d (Vitacost Vitamin D_3_, Boca Raton, FL, USA) or 7000IU/d (Life Extension, Ft. Lauderdale, FL, USA) D_3_ tablets. Doses were verified (Tampa Bay Analytical Research, Largo, FL, USA). The randomization sequence assigned subjects in a 1:1 ratio within each age group, was generated using STATA 12.0 (College Station, TX) and given to a research pharmacist who labeled the medications. Clinicians, investigators and participants were blinded to randomization. Adherence was assessed by pill count from returned bottles and determined as a percent.

### Assessment of outcome

Efficacy was defined as serum 25D ≥32ng/mL at 12-weeks; safety as a low incidence (<5%) of subjects with a simultaneously elevated serum calcium (using age and sex specific ranges) and 25D (>160ng/mL). Nurses interviewed participants after 2 weeks and at each visit for the occurrence of adverse events. Laboratory results were reviewed within 24 hours.

### Statistical analysis

All variables were tested for normality and nonparametric tests were used as appropriate. Means, medians and other parameters as appropriate described outcome variables at study visits. Differences between groups at baseline or over time were assessed using unpaired t tests or Mann Whitney U tests as appropriate and chi-squared tests for categorical variables. Significance of change from baseline over time was assessed using longitudinal mixed effects (LME) models adjusting for baseline values, age and sex. Age group and ART regimen were explored as effect modifiers of 25D change (Δ25D). Models were adjusted for potential predictors of 25D including baseline 25D, sex, adherence, season, and years of ART, with age group x time or ART regime x time interaction terms for differences between groups in Δ25D. The five age groups used for enrollment stratification were collapsed to 3 groups for analyses for children (5–13y), adolescents/young adults (14–29y) and older adults (30–50y). Statistical analyses were performed using STATA 12.0 (College Station, TX), and results considered significant at p<0.05.

### Ethics statement

Ethics Boards of the Botswana Ministry of Health, Princess Marina Hospital, Baylor College of Medicine, the University of Pennsylvania and CHOP approved the study. Adults gave written informed consent. For those under 21 years, a legal guardian provided written informed consent, and written assent was given by those 7 to 21 years. Due to an oversight error, this trial was not registered in a clinical trials registry before it was completed—it was however registered on clinicaltrials.gov before manuscript submission (ClinicalTrials.gov Identifier: NCT02189902). The authors confirm that all ongoing and related trials for this drug/intervention are registered.

## Results

**Fig 1:**
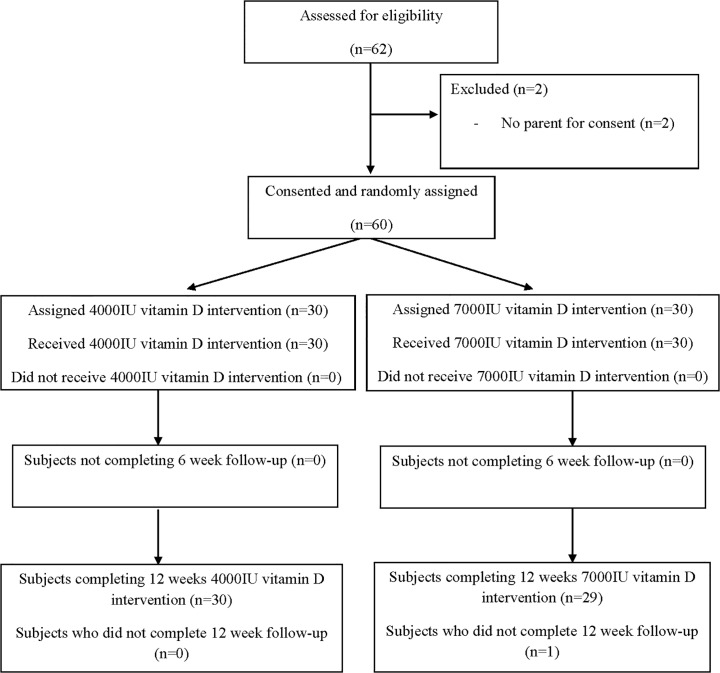
Consort flow diagram for subjects randomized, drop-outs, and completing the trial of daily 4000IU or 7000IU vitamin D3 supplementation in HIV-infected children and young adults.

After prescreening, 62 subjects were assessed for eligibility and 60 subjects were enrolled (Fig. 1 shows the consort diagram). Of 60 subjects enrolled, 30 received 4000IU/d and 30 received 7000IU/d D_3_, evenly by age group. Fifty-nine completed all study visits. Subjects were 50% male, 68% perinatally infected, age 19.5±11.8 years (mean±SD) with predominantly moderate to severe AIDS defining illness (Table 1). At enrollment, 23 to 43% of those under 20 years had height adjusted Z score (HAZ), weight adjusted Z score (WAZ) or body mass index Z score (BMIZ) more than two standard deviations below the reference median. More males were in the 7000IU/d group, but groups did not otherwise differ at baseline. The mean (SD) duration of ART for the whole cohort was 5.0 years (±2.7) and did not differ between D_3_ dose groups.

**Table 1:** Characteristics of Subjects at Baseline by D_3_ Dose Group.

	All	4,000 IU	7,000 IU
N	60	30	30
Age, y	19.5 ± 11.8	19.5 ± 11.8	19.5 ± 12.0
Sex, % male	50	37	63*
Perinatally acquired ^a^, %	68	66	70
Season, %			
Summer: Nov—Jan	37	33	40
Autumn: Feb—Apr	63	67	60
Pediatric nutritional status (n = 40) ^b^			
Height Z score≤2, %	28	35	20
Weight Z score≤2, %	43	45	40
BMI Z score≤2, %	23	20	25
BMI > 85^th^ percentile, %	3	1 (5	0 (0)
Adult nutritional status (n = 20) ^b^			
Height, cm	163.0 ± 9.4	162.0 ± 10.9	164.1 ± 8.2
Weight, kg	58.3 ± 13.9	59.4 ± 18.3	57.2 ± 8.4
BMI, kg/cm^2^	22.0 ± 5.8	22.8 ± 7.7	21.3 ± 3.4
BMI<18, %	20	10	10
BMI>30, %	5	10	0
BMI>25, %	20	30	10
Years since HIV diagnosis ^c^	6.2 ± 2.7	6.5 ± 2.5	5.8 ± 2.9
Duration of ART treatment ^d^, y	5.0 ± 2.7	5.2 ± 2.6	4.9 ± 2.8
CDC HIV Classification ^e^ (%)			
N	14	14	14
A	20	21	18
B	11	11	11
C	55	54	57
Immunity category: worst (%)			
CD4 count ≥ 500	29	22	35
CD4 count 200–499	34	44	24
CD4 count < 200	38	33	41
Immunity category: current (%)			
CD4 count ≥ 500	68	81	55
CD4 count 200–499	27	19	34
CD4 count < 200	5	0	10
CD4 ^f^ %	31.8 ± 10.4	33.8 ± 8.3	30.0 ± 11.9
Viral load, RNAlog ^g^	1.54 ± 0.42	1.44 ± 0.18	1.64 ± 0.56
Viral load, detectable %	19	12	26
ART Regimens, %			
PI	25	27	23
NNRTI: ALL	75	73	77
Efavirenz	33	30	37
Nevirapine	42	43	40
Tenofovir^h^	13	17	10
& NNRTI	13	17	10
& PI	0	0	0

*Dose groups significantly different p<0.05

^a^ n = 59, one subject had unknown HIV acquisition

^b^ n = 40 for subjects <20y with calculated Z scores for body size variables (20 in 4000 and 20 in 7000IU/d group) n = 20 for subject ≥20y (10 in 4000 and 10 in 7000IU/d group)

^c^ n = 54 subjects with date of diagnosis for HIV (28 in 4000 and 26 in 7000IU/d group)

^d^ n = 59 subjects with date of initiation of ART treatment (30 in 4000 and 29 in 7000IU/d group)

^e^ n = 56 subjects with HIV CDC classification data and CD4 counts from medical record review (27 in 4000 and 29 in 7000IU/d group)

^f^ n = 59 with CD4% baseline (29 in 4000 and 30 in 7000IU/d group)

^g^ n = 53 RNA viral load at baseline (26 in 4000 and 27 in 7000IU/d group)

^h^ Tenofovir was used only in combination with NNRTI-based regimens.

HIV, human immunodeficiency virus; BMI, body mass index; CDC, Center for Disease Control; Clinical categories: N = asymptomatic, A = mildly symptomatic, B = moderately symptomatic, and C = severely symptomatic with AIDS defining illness; CD4 cluster of differentiation; PI, protease inhibitor-based anti-retroviral treatment (ART); NNRTI, non-nucleoside reverse transcriptase-based ART.

**Fig 2:**
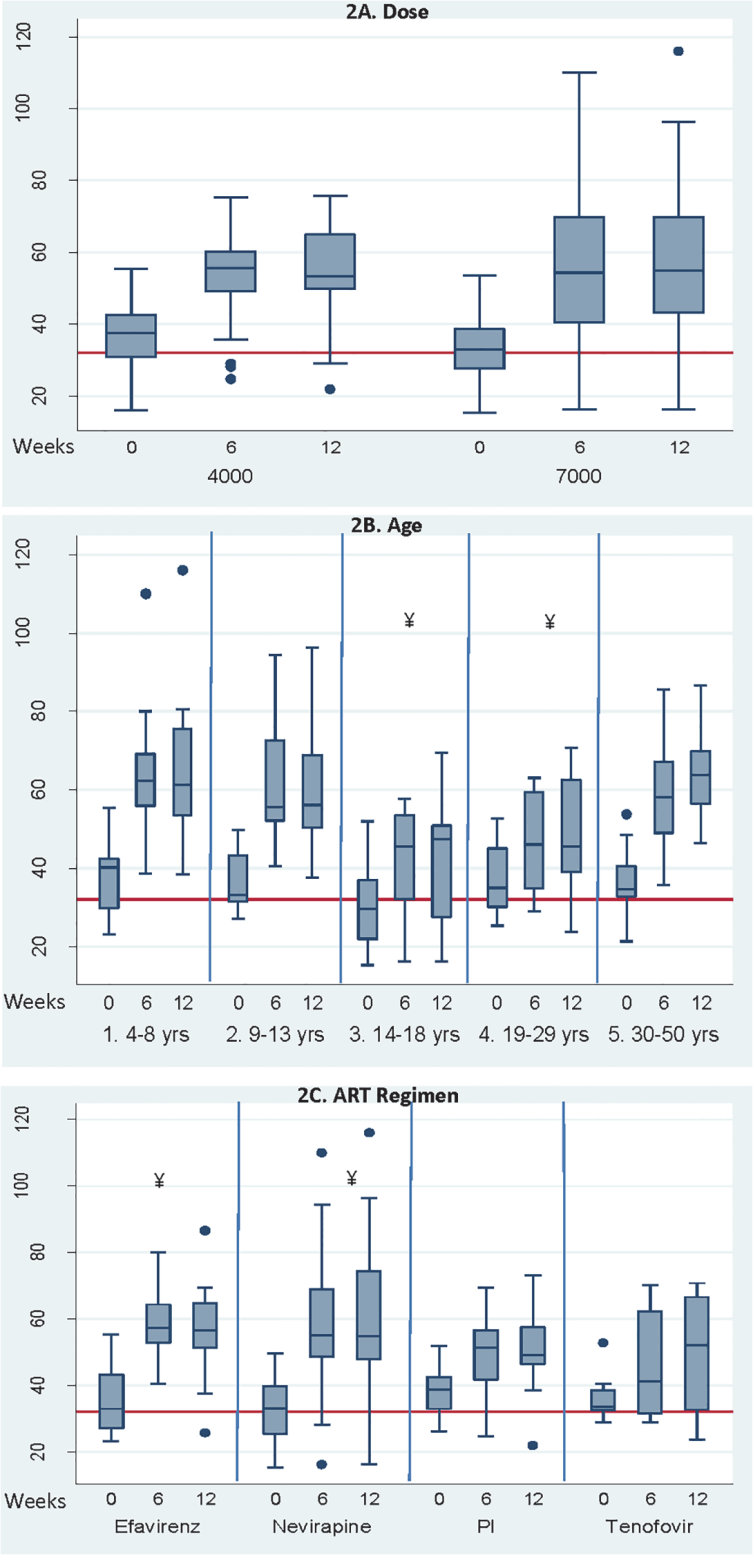
Serum 25D Before and After High Dose D_3_ Supplementation. **A**. By Dose group (4000 IU/d or 7000IU/d). * Change in 25D significantly different from baseline at both 6 and 12 weeks, p<0.01. **B**. By age group. ¥ Change in 25D significantly less in subjects ages 14–29 y than those ages 4–13 y and 30–50 y age groups, p<0.004. **C**. By anti-retroviral therapy (ART) regime. ¥ Change in 25D significantly greater in Efavirenz and Nevirapine groups than in PI (protease inhibitor) or tenofovir groups, p<0.03

D_3_ supplementation was effective as both serum 25D and bioavailable 25(OH)D increased from baseline in more than 80% of the participants (Table 2). The proportion of bioavailable to serum 25D did not change. Using LME models adjusted for baseline 25D, age, and sex, Δ25D at 6 and 12-weeks of treatment did not differ between groups (Fig. 2A). Two subjects at 6 weeks and two at 12 weeks (total 3 subjects), all in the 7000IU/d dose group, had 25D levels >90ng/ml [19].

**Table 2:** Clinical and laboratory values for subjects over time by D_3_ dose group.

	Baseline		6 weeks		12 weeks	
	4,000 IU	7,000 IU	4,000 IU	7,000 IU	4,000 IU	7,000 IU
N	30	30	30	30	30	29
Total 25(OH)D, ng/ml	36.5 ± 9.3	34.5 ± 9.5	53.4 ± 11.7***	56.4 ± 21.0***	54.8 ± 13.0***	56.5 ± 22.4***
≤ 20 ng/ml, %	3	7	0	3	0	3
20–31 ng/ml, %	23	30	10	3	7	10
≥ 32 ng/ml, %	73	63	90	94*	93	87
Bioavailable 25(OH)D, ng/ml ^a^	9.4 ± 2.7	8.5 ± 2.6	-	-	15.7 ± 6.1***	16.1 ± 8.2***
Bioavailable/Total 25(OH)D, % ^a^	26.1 ± 3.7	26.2 ± 4.9	-	-	27.9 ± 7.0	29.1 ± 6.4
1,25D, pg/ml	66.8 ± 33.1	57.2 ± 18.8	-	-	72.0 ± 34.0	71.1 ± 27.5***
PTH, pg/ml±	33.6 ± 18.9	29.8 ± 17.0	-	-	25.6 ± 10.4**	24.4 ± 13.7*
DBP, umol/L ^a^	1.8 ± 0.4	1.7 ± 0.5	-	-	1.8 ± 0.7	1.7 ± 0.8
Magnesium, mmol/L	1.0 ± 0.1	0.9 ± 0.1	0.9 ± 0.1	1.0 ± 0.2	0.9 ± 0.1*	0.9 ± 0.1
Phosphorous, mmol/L	1.3 ± 0.3	1.3 ± 0.3	1.2 ± 0.2	1.3 ± 0.3	1.2 ± 0.3	1.3 ± 0.3
Calcium, mmol/L	2.3 ± 0.1	2.3 ± 0.1	2.3 ± 0.1	2.3 ± 0.1	2.3 ± 0.1	2.3 ± 0.1
Albumin, g/L	39.7 ± 3.3	38.9 ± 3.9	39.3 ± 3.4	38.9 ± 3.6	39.8 ± 3.2	39.5 ± 3.5
Corrected Calcium, mmol/L	2.3 ± 0.1	2.3 ± 0.1	2.3 ± 0.1	2.3 ± 0.2	2.3 ± 0.1	2.3 ± 0.1
Blood Lead, μg/dl	2.2 ± 1.3	2.6 ± 2.6	2.4 ± 2.1	3.2 ± 5.3	2.8 ± 2.5*	2.8 ± 3.2
CD4 ^b^, mm^3^	820 ± 421	758 ± 540	__	__	765 ± 334	693 ± 451*
CD4, %	33.2 ± 8.0	29.7 ± 12.0	__	__	33.4 ± 7.2	31.5 ± 12.6**
Viral load ^c^, copies/mL	34 ± 39	367 ± 1324	__	__	776 ± 3348	351 ± 1478
Viral load, RNA log	1.46 ± 0.20	1.70 ± 0.62	__	__	1.57 ± 0.62	1.55 ± 0.54*
Viral load, % detectable ≥25	15	29	__	__	15	14
Height Z Score ^d^	-1.38 ± 1.14	-1.48 ± 1.02	-1.39 ± 1.16	-1.42 ± 1.02*	-1.38 ± 1.13	-1.42 ± 1.03**
Weight Z score	-1.60 ± 1.53	-1.84 ± 1.42	-1.61 ± 1.57	-1.84 ± 1.42	-1.50 ± 1.49*	-1.73 ± 1.42*
BMI Z score	-1.12 ± 1.48	-1.20 ± 1.16	-1.13 ± 1.51	-1.28 ± 1.12	-0.99 ± 1.43*	-1.11 ± 1.09

*Significantly different from baseline within the dose group at p<0.05 (LME model adjusted for age, sex and baseline value)

**p≤0.01

***p<0.001

^a^ n = 49, Bioavailable 25(OH)D and DBP at baseline (24 at 4000 and 25 at 7000IU/d D_3_) and n = 51 12 weeks visit (26 at 4000 and 25 at 7000IU/d D_3_)

^b^ n = 56, CD4 baseline and 12 weeks visit (27 at 4000 and 29 at 7000IU/d D_3_)

^c^ n = 41, RNA viral load analysis baseline and 12-week visit (20 at 4000 and 21 at 7000IU/d vit D_3_)—as these 41 subjects had VL run at the same laboratory both times—baseline and 12 weeks

^d^ n = 40 subjects <20y with calculated Z scores for body size variables (20 in 4000 and 20 in 7000IU/d group). HIV, human immunodeficiency virus; BMI, body mass index

CD4, cluster of differentiation; PTH, parathyroid hormone; DBP, vitamin D binding protein.

The increase in 25D with D_3_ supplementation was associated with decreased PTH (both groups), increased 1,25D in the 7000IU/d group and no change in DBP. WAZ and BMIZ increased in those under 20 years in both groups, and HAZ increased in the 7000IU/d group. For subjects older than 20 years, BMI increased 0.4 units at 12 weeks (p = 0.02).

Adherence to D_3_ was 84±17%; and did not differ by dose—4000 and 7000IU/d dose group’s adherence was 86±15% vs. 81±15%, respectively. D_3_ supplementation was safe with no simultaneous elevations in serum calcium and 25D or adverse clinical or laboratory events recorded. There was a 5% incidence of high serum calcium at 6 weeks (2/30 [7%] and 1/30 [3%] in the 4000 and 7000 IU/d dose groups, respectively) which required no clinical intervention. The groups did not differ in the incidence of high serum calcium and there was no one with high serum calcium at either baseline or 12 weeks. Outcomes did not differ by HIV acquisition.

Both age group and ART regimen were significant effect modifiers of 25D response to D_3_ supplementation (Fig. 2B and 2C, respectively). From unpaired t tests, younger children (5 to 13 years) and older adults (30 to 50 years) had greater Δ25D than adolescents and younger adults (14 to 29 years), with Δ25D of 26±17, 28±12 and 11±11ng/ml, respectively (p≤0.001) (Fig. 1B). Using LME models adjusted for baseline 25D, sex, adherence, season, and years of ART, with age group x time interaction, the 14 to 29 year age group had a lower Δ25D than the other age groups (Coefficient [95% confidence intervals], -1.3ng/mL/wk [-2.0,-0.6], R^2^ = 0.60, p<0.001). Only 75% of adolescents and young adults achieved 25D >32ng/mL compared to 100% of younger and older groups (p<0.05). Adherence did not differ between age groups (81 to 87%) or ART regimen (82 to 85%) and did not explain differences in Δ25D in any model.

For ART regimen (Fig. 2C), Δ25D was two-fold higher in subjects receiving efavirenz (22±12) or nevirapine (27±17) compared to those receiving PIs (13±10) (p≤0.03). At 6 weeks, both NNRTI regimens resulted in greater Δ25D than those receiving PIs (p<0.03). LME models adjusted for baseline 25D, age, sex, adherence, season and years of ART, with ART regimen x time interaction, showed a greater Δ25D in NNRTI regimens compared to PIs (0.8ng/mL/wk [0.1,1.5], R^2^ = 0.57, p<0.044). Those on nevirapine experienced significantly greater Δ25D over time than those treated with PI-based regimens (1.1ng/mL/wk [0.3, 2.0] R^2^ = 0.58, p = 0.007). Subjects receiving tenofovir (n = 8, all on NNRTI regimens) did not differ in Δ25D from subjects on other regimens.

## Discussion

In this pilot study, daily 4000 and 7000 IU D_3_ supplementation over 12 weeks was safe and improved vitamin D status. Age and ART regimen modified the response, and mode of HIV acquisition did not. Of note, weight status improved in children and adolescents receiving either dose and height status improved in those receiving 7000IU/d after 12-weeks. Both doses resulted in more than 80% of subjects achieving a 25D >32ng/mL. These high vitamin D doses were safe with no treatment-related clinical or laboratory adverse events. Previous pediatric and young adult vitamin D supplementation studies were based in North America and utilized lower doses and longer dose intervals than used here [20,21,22].

In the present study, vitamin D supplementation was associated with improved weight and BMI status in children and adolescents, with improved height in those on 7000IU/d. BMI increased in adults (20 to 50 years), despite the relatively short 12-week duration. Other vitamin D studies of children with HIV have either not reported on growth [20,21] or did not find a change [22,23]. The findings of improved WAZ and BMIZ with both D_3_ doses and HAZ in the 7000IU/d group were unexpected. However, the 12-week increase of 0.10 and 0.11 in weight and WAZ or BMIZ, if sustained, represents a change of 0.4 to 0.5 Z scores/year, a potentially clinically significant improvement.

Ganmaa et al [24] showed an increase in height with D_3_ supplementation in 120 Mongol school children (12–18y) who received 800IU/d D_3_ compared to placebo for six months encompassing winter. In the same region [25], a vitamin D-fortified whole milk-based supplement in 46 children (9 to 11 years) demonstrated an increase in linear growth over one-month. A seasonal growth pattern in healthy children has been demonstrated, with increased growth in sunny seasons, both in resource-rich and resource-limited environments, and a vitamin D mechanism has been postulated [26,27]. Our Batswana sample with relatively poor baseline growth status is among the first to show improvement in growth status with D_3_ supplementation in children with HIV. Although seasonal growth resulting from seasonal variations in food supply or infectious burden has been reported from other African settings [28], this has not been described in Botswana and the effect of season was insignificant in our analyses. Change in 25D was significantly less in our adolescents and young adults than in children or older adults. This finding was not explained by differences in sex, adherence, ART regimen, or time on ART.

Several studies have documented reduced vitamin D status in people with HIV treated with NNRTIs [2,4,5,20,29,30]. Our study is the first to document this in an African setting. Little is known regarding the effect of ART regimen on response to D_3_ supplementation. A 12-week D_3_ supplementation study of American youth (18–24y) found that efavirenz did not diminish response to D_3_ despite being associated with poorer vitamin D status at baseline [20]. In our sample, children and adults treated with an NNRTI did not have poorer vitamin D status at baseline, however, they had a significantly larger change in 25D with high dose D_3_ supplementation than those on PIs. The vitamin D response for the eight subjects on tenofovir plus NNRTIs did not differ from those on non-tenofovir based regimens. The more blunted response in those on PIs may suggest a possible alteration in metabolism of D_3_. Powe et al [31] demonstrated the importance of DBP status in black compared to white Americans with black participants having lower DBP and 25D, but similar bioavailable 25(OH)D. In our Botswana African sample, the DBP was even lower (1.8 μmol/L) than that seen in American blacks in the Powe et al. study (3.3 μmol/L) [31].

This pilot study included a number of limitations. The small sample size and single site experience limits generalizability and requires further study in multisite samples. Without a control group, it is not possible to evaluate if the changes measured were attributable to the intervention or were the positive effect of enrollment in the study. The mixed age population and the mixed background ART add heterogeneity to the study population and were accounted for in the analysis. To assess the effects on growth, a future study should only target those under 20 years of age.

Micronutrient supplementation which did not contain vitamin D reduced the risk of immune status decline and morbidity in ART-naïve HIV-infected adults in Botswana [32]. Our results in this pilot study suggest that high dose vitamin D supplementation is safe in the African setting. Additionally, the 7,000IU/d may convey a growth in height advantage in children and adolescents. Further study with a larger sample size and including a control group is warranted to understand mechanisms underlying differences between ART regimens in response to D_3_ supplementation as well as to explore the growth and age responses.

## Supporting Information

S1 CONSORT Checklist Study CONSORT checklist. (DOC)

S1 Protocol Study protocol version November 22^nd^, 2010. (PDF)

S2 Protocol Study protocol version December 14^th^, 2011. (PDF)

S3 Protocol Study protocol version March 19^th^, 2012. (PDF)

S4 Protocol Study protocol version May 17^th^, 2012. (PDF)

S1 Study Dataset Dataset for this study in Excel2007 format. (XLSX)

S2 Study Dataset Dataset for this study in PDF format. (PDF)
