# Supplementary material for: Vitamin D₃ Supplementation in Batswana Children and Adults with HIV: A Pilot Double Blind Randomized Controlled Trial
Source: PLoS One. 2015 Feb 23;10(2):e0117123. doi: 10.1371/journal.pone.0117123 (PMC4338235; doi:10.1371/journal.pone.0117123)
Supplement: S1 Protocol — (PDF) [file pone.0117123.s002.pdf]

Botswana Vitamin D Supplementation Study in HIV/AIDS

Principal Investigators:

Virginia Stallings, MD
Director, Nutrition Center
The Children's Hospital of Philadelphia

Other Investigators:

Name:	Organization:	Email:	Telephone Number:
Andrew Steenhoff, MD	Children's Hospital of Philadelphia	Steenhoff@email.chop.edu	(+267) 3170933
Richard Rutstein, MD	Children's Hospital of Philadelphia	rutstein@email.chop.edu	(+1)215-590-1466
Babette Zemel, PhD	Children's Hospital of Philadelphia	zemel@email.chop.edu	(+1)215-590-1686
Joan Schall, PhD	Children's Hospital of Philadelphia	schall@email.chop.edu	(+1)215-590-5688
John Pettifor, PhD	University of Witwatersrand	John.Pettifor@wits.ac.za	27-11-933-1530
Loeto Mazhani, MD	Princess Marina Hospital	lomazhani@yahoo.com	(+267) 71677145
Michael Tolle, MD	Botswana-Baylor Children's Clinical Centre of Excellence	mtolle@baylorbotswana.org.bw	(+267)319-0083
Maria Nnyepi, PhD	University of Botswana	nnyepims@mopipi.ub.bw	(+267) 3552447
Unabatsho Maposa, MD	Princess Marina Hospital	jibitjibi@yahoo.co.uk	(+267) 724 72797
Bakgaki Ratshaa	Botswana-UPenn Partnership	bakgaki@gmail.com	(+267) 71613100
Phillip Pusoesele	Botswana-UPenn Partnership	pusoeselep@botswana-upenn.co.bw	(+267) 727 26706

A. Specific Aims:

To test **two oral daily doses** (4000 vs. 7000 IU) of cholecalciferol (D3) dietary supplement over a 12-week period in 60 children and adults with HIV/AIDS living in Botswana (5.0 to 50.9 yrs), to assess **safety** as determined by serum calcium and 25D concentrations and **efficacy** to replete vit D status as determined by achieving a minimum serum 25D concentration of 32 ng/mL (80 nmol/L). Cathelicidin antimicrobial protein activity will be used as an indicator of immune response to vit D depletion and repletion.

B. Background:

The vit D system consists of metabolites derived from vit D_2 (ergocalciferol) and D_3 (cholecalciferol). Vit D_3, the more common circulating form, is derived from dietary sources or synthesized in the skin after exposure to sunlight. Although a major function of vit D is to maintain calcium homeostasis and bone mineralization, the discovery of many cells that express 25(OH)D 1-α-hydroxylase activity (macrophages, breast, colon, pancreas, brain, ovary, dendritic and endothelial cells) has established the other major physiological roles[1]. Vit D deficiency has been implicated in type 1 and type 2 diabetes, cancer, cardiovascular disease, obesity, and multiple sclerosis, suggesting anti-proliferative, immunomodulatory, anti-inflammatory and anti-cancer effects of vit D[1;2].

Vit D deficiency and health: The best indicator of vit D stores is the serum concentration of $25D_3$. When circulating 25D concentrations are inadequate, intestinal calcium absorption and bone mineralization are impaired. Vit D deficiency leads to a decrease in serum ionized calcium, initiating increased synthesis and secretion of parathyroid hormone (PTH) to normalize calcium homeostasis. When prolonged, this results in skeletal demineralization (rickets or osteomalacia) and myopathy[3;4]. Vit D deficiency has been linked to other health effects as well, such as increased blood pressure (BP), diabetes, and cardiovascular complications.

Vit D also fights infections by increasing antimicrobial peptides such as β-defensins and cathelicidins that kill bacteria, viruses, fungi and protozoa and are produced by mucosal and circulating white cells[5;6], and vit D_3 may play an important role in both prevention of urinary tract infections and protection of skin wounds against infection. Cathelicidin (LL-37) is one antimicrobial peptide that may work in this way, and the cathelicidin gene is markedly upregulated by 1,25D. Studies among West Africans[7], Tanzanians[8], and among African immigrants to Australia[9] have shown that higher serum 25D is associated with a lower probability of M. tuberculosis infection. Conversely, individuals with TB or a past history of TB are significantly more likely to be vit D deficient than those without the disease (African migrants to Australia[9]). Vit D may enhance immunity to M. tuberculosis through the vit-D dependent production of cathelicidin[10-13].

Therefore, the role of vit D supplementation in preventing TB infection may have particular salience for people living with HIV/AIDS in African countries such as Botswana and worldwide. Circulating monocytes and antigen presenting cells (dendritic cells, macrophages) have the VDR and hydroxylase enzymes and convert 25D to 1,25 D locally. Also the innate immune response includes activation of the Toll-like receptors (TLR) of human immune cells and directs the antimicrobial activity for intracellular bacteria. The TLR system is sensitive to vit D deficiency and acts through the cathelicidin peptide pathway[5;14]. We will use the cathelicidin system as a 25D biomarker and follow the cathelicidin response to D_3 supplementation.

Assessment of vit D status: In adults, functional measures have been used to define vit D adequacy as a serum 25D level greater than 32 ng/mL (80 nmol/L): vit D insufficiency is commonly defined as 25D levels between 10-30 ng/mL, vit D deficiency <10 ng/mL and high risk for vit D toxicity, 25D level >160 ng/mL[15;16].

Prevalence of vit D deficiency: Vit D deficiency remains an under-recognized problem in the general population and is poorly defined in children. Inadequate circulating 25D has been documented in numerous surveys of children and adults. Blacks have greater risk for vit D deficiency[17], largely due to the decreased ability of pigmented skin to synthesize vit D[18-20]. When cutaneous production of vit D is impaired, adequate dietary intake is required. Dietary intake is usually inadequate since few foods (fish oils, egg yolks) naturally contain vit D.

Vit D insufficiency and rickets have been found to be prevalent in African countries, despite adequate sunlight exposure. The proportion of individuals with insufficient vit D status (<32 ng/mL) in Gambia was found to be 23% among children ages 8 to 12 yr, and 55% in young women (<45 yr)[21]. Among 884 HIV-infected pregnant women living in Tanzania, 39% had low vit D (<32 ng/mL), and these women had more rapid HIV disease progression, higher all-cause mortality and a higher incidence of anemia, than women with optimal vit D status (≥32 ng/mL)[22]. Furthermore, the children born to women with low vit D status had a 46% higher risk of HIV infection and a 61% higher risk of dying in the first 24 months of life[23]. Pettifor has suggested that the high prevalence of rickets in African countries may be, in part, the result of low calcium intake in these countries which may increase requirements for vit D[24].

Vit D status and HIV in adults: Poor vit D status has been documented in both adults and children with HIV infection in both developed and developing countries. The reason for low vit D status in people living with HIV is not understood, however contributing factors include inadequate sunlight exposure, low dietary and supplement intake, increased utilization, or increased loss through malabsorption. Despite advances in our understanding of the potent immunomodulatory activity of vit D, its role in HIV disease progression is largely unknown[25].

Vit D status and HIV disease severity: Vit D affects the development and function of cells of the immune system that help control HIV infection, including macrophages and T lymphocytes[26], and is known to stimulate monocyte differentiation[25]. 1,25D may enhance macrophage function in subjects with HIV[27], or may have a direct effect upon decreasing bacteria replication in the HIV-infected host[28]. Studies have found a positive association between CD4+ cell counts and serum 25D,[29-31], and dietary intake of vit D[32].

Vitamin D Dose Selection: The doses of vit D_3 chosen for this study are based upon evidence from several studies: 1) Heaney et al[33] suggest that vit D_3 from all sources of 3600 to 4200 IU/d are required to sustain 25D concentrations of >80 nmol/L (>32 ng/mL) in adults; 2) Aloia et al[34] found that a mean daily dose of 3440 IU was necessary to attain 25D concentrations >30 ng/mL in a sample of African American and white healthy adults living in New York, and those with baseline values <22 ng/mL required 5000 IU; and 3) Vieth et al[35] demonstrated that a dose of 100 ug/d (4000 IU/d) of vit D_3 resulted in a minimum serum 25D plateau concentration of 69 nmol/L in adults. Because we expect that subjects with HIV living in Botswana may require higher levels of vit D supplementation due to the varied HIV medications they take, their likely low dietary intake of vit D, and their darker skin pigmentation, we propose doses of 4000 and 7000 IU/day to insure that nearly all subjects will achieve serum 25D concentrations >80 nmol/L (>32 ng/mL).

Safety of Vitamin D Supplementation: Reports of vit D toxicity are very rare and generally associated with intakes >30,000 IU/d due to either industrial accidents or unintentional overdoses of unregulated concentrated vit D supplements. There have been no reports of vit D toxicity from typical dietary intake or sunlight exposure[36]. Our primary safety outcome is serum hypercalcemia (age-adjusted range) associated with 25D concentrations >160 nmol/L. We will also assess serum albumin, magnesium and phosphorus, urinary calcium, and ask questions to elicit the subject and family report of new signs and symptoms of adverse events. Manifestations of hypercalcemia associated with elevated 25D are anorexia, nausea, vomiting, weakness, lethargy, constipation, polyuria, polydypsia and general disorientation[37].

Summary: In summary, many people living with HIV/AIDS in African countries are vit D deficient or insufficient. Vit D deficiency in HIV/AIDS may be due to low dietary vit D intake, increased requirements, malabsorption, specific drug therapies (antiretrovirals, in particular), reduced outdoor physical activity, reduced vit D synthesis from UV light exposure in dark skin pigmented individuals, or unknown HIV-associated factors. Vit D deficiency likely contributes to abnormal immune status and increased inflammatory state, and to poor growth, bone, and muscle function, and may contribute to risk for TB infection. The goal is to determine the vit D supplementation dose that safely results in optimal serum 25D concentrations in HIV-infected children and adults living in Botswana.

C. Study Design:

To test two oral doses (4000 vs 7000 IU/d) of cholecalciferol (D_3) over a 12-week period in 60 children and adults (5.0 to 50.9 y) with HIV/AIDS to assess ***safety*** as determined by serum calcium and 25D concentrations and ***efficacy*** to replete vit D status as determined by achieving a minimum serum 25D concentration of 32 ng/mL. The response of cathelicidin antimicrobial protein activity to vit D_3 supplementation will also be assessed as an indicator of immune response to vit D *depletion and repletion*. We will enroll the 60 subjects within 5 different age groups, based upon Dietary Reference Intakes. Twelve subjects will be recruited in each of the following six age categories (5.0-8.9, 9.0-13.9, 14.0-18.9, 19.0-30.9, 31.0-50.9 yr), and will be randomized to receive either 4000 IU/d or 7000 IU/d vit D_3. Every attempt will be made for equal gender representation.

<u>Vit D Supplement:</u> Given the varied medications and likely low dietary intake of vit D in subjects with HIV/AIDS, we propose doses of 4000 and 7000 IU/d in this sample children and adults in Botswana to assure that all, or nearly all subjects will achieve serum 25D concentrations >32 ng/mL. 2000 IU and 5000 IU tablets of vit D_3 (cholecalciferol) will be provided. Subjects will take two 2000 IU tablets per day, or one 5000 IU tablet and one 2000 IU tablet per day depending on whether they are randomized to receive 4000 or 7000 IU/day vit D_3. Drops will be provided to subjects who experience difficulty swallowing tablets, either four drops of 1000 IU/drop or seven drops of 1000 IU/drop depending upon randomization group. All research personnel will be blinded to the randomization throughout the study.

Inclusion Criteria:

- HIV infection
- Ages 5.0 to 50.9 y
- In usual state of good health
- Subject and/or family commitment to the 12-week study

Exclusion Criteria:

- Other chronic health conditions unrelated to HIV/AIDS that may affect nutritional status

- Use of vit D supplementation above 400 IU/d

Sample Size

30 subjects at each of the 2 dose levels (4000 IU/d and 7000 IU/d) will be enrolled. We will enroll 12 subjects in each of 5 age groups (5.0-8.9, 9.0-13.9, 14.0-18.9, 19.0-30.9, 31.0-50.9 yrs), 5 subjects at each of the 2 dose levels within each age group. This is pilot work to explore the efficacy of each dose level in repleting 25D levels to ≥32 ng/mL after 12 weeks of supplementation and in increasing mRNA expression in the cathelicidin pathway by 100%. This will establish feasibility and provide preliminary data for study design and sample size calculations for future studies of vit D supplementation in Botswana.

Logistics and Timeline

Subject recruitment will begin January 10, 2011 and enrollment will end April 8, 2011. 6 week visits will begin February 21, 2011 and conclude May 20, 2011. 12 week visits will begin April 4 and end July 1. The month of July will be dedicated to data analysis. The study will be complete by August 2011.

Limitations

Enrollment and retention are the most likely difficulties and limitations in a nutrition intervention study. Enrollment is expected to progress as scheduled based upon the experience in the UPenn/Botswana research team.

Confidentiality, Subject Risks, and Benefits

All data will be recorded in a password-controlled database. Data collection forms will be stored in a locked cabinet behind a locked door and only the study team will have access to them. This study poses no physical risk to subjects. There is a small risk of loss of confidentiality; however, subject names will not be collected and only study investigators will have access to the forms.

Reference List

(1) Townsend K, Evans KN, Campbell MJ, Colston KW, Adams JS, Hewison M. Biological actions of extra-renal 25-hydroxyvitamin D-1alpha-hydroxylase and implications for chemoprevention and treatment. J Steroid Biochem Mol Biol 2005; 97:103-109.

(2) Holick MF. Vitamin D: importance in the prevention of cancers, type 1 diabetes, heart disease, and osteoporosis. Am J Clin Nutr 2004; 79(3):362-371.

(3) Heaney RP. Lessons for nutritional science from vitamin D. Am J Clin Nutr 1999; 69(5):825-826.

(4) Pfeifer M, Begerow B, Minne HW. Vitamin D and muscle function. Osteoporos Int 2002; 13(3):187-194.

(5) Zasloff M. Fighting infections with vitamin D. Nat Med 2006; 12(4):388-390.

(6) Adams JS, Liu PT, Chun R, Modlin RL, Hewison M. Vitamin D in defense of the human immune response. Ann N Y Acad Sci 2007; 1117:94-105.

(7) Wejse C, Olesen R, Rabna P, Kaestel P, Gustafson P, Aaby P et al. Serum 25-hydroxyvitamin D in a West African population of tuberculosis patients and unmatched healthy controls. Am J Clin Nutr 2007; 86(5):1376-1383.

(8) Friis H, Range N, Pedersen ML, Molgaard C, Changalucha J, Krarup H et al. Hypovitaminosis D is common among pulmonary tuberculosis patients in Tanzania but is not explained by the acute phase response. J Nutr 2008; 138(12):2474-2480.

(9) Gibney KB, MacGregor L, Leder K, Torresi J, Marshall C, Ebeling PR et al. Vitamin D deficiency is associated with tuberculosis and latent tuberculosis infection in immigrants from sub-Saharan Africa. Clin Infect Dis 2008; 46(3):443-446.

(10) Liu PT, Stenger S, Tang DH, Modlin RL. Cutting edge: vitamin D-mediated human antimicrobial activity against Mycobacterium tuberculosis is dependent on the induction of cathelicidin. J Immunol 2007; 179(4):2060-2063.

(11) Adams JS, Chen H, Chun R, Ren S, Wu S, Gacad M et al. Substrate and enzyme trafficking as a means of regulating 1,25-dihydroxyvitamin D synthesis and action: the human innate immune response. J Bone Miner Res 2007; 22(S2):V20-V24.

(12) Liu PT, Modlin RL. Human macrophage host defense against Mycobacterium tuberculosis. Curr Opin Immunol 2008; 20(4):371-376.

(13) Adams JS, Ren S, Liu PT, Chun RF, Lagishetty V, Gombart AF et al. Vitamin d-directed rheostatic regulation of monocyte antibacterial responses. J Immunol 2009; 182(7):4289-4295.

(14) Liu PT, Stenger S, Li H, Wenzel L, Tan BH, Krutzik SR et al. Toll-like receptor triggering of a vitamin D-mediated human antimicrobial response. Science 2006; 311(5768):1770-1773.

(15) Dawson-Hughes B, Heaney RP, Holick MF, Lips P, Meunier PJ, Vieth R. Estimates of optimal vitamin D status. Osteoporos Int 2005; 16(7):713-716.

(16) Heaney RP. Vitamin D: criteria for safety and efficacy. Nutr Rev 2008; 66(10 Suppl 2):S178-S181.

(17) Nesby-O'Dell S, Scanlon KS, Cogswell ME, Gillespie C, Hollis BW, Looker AC et al. Hypovitaminosis D prevalence and determinants among African American and white women of reproductive age: third National Health and Nutrition Examination Survey, 1988-1994. Am J Clin Nutr 2002; 76(1):187-192.

(18) Holick MF, MacLaughlin JA, Doppelt SH. Regulation of cutaneous previtamin D3 photosynthesis in man: skin pigment is not an essential regulator. Science 1981; 211(4482):590-593.

(19) Clemens TL, Adams JS, Henderson SL, Holick MF. Increased skin pigment reduces the capacity of skin to synthesise vitamin D3. Lancet 1982; 1(8263):74-76.

(20) Armas LA, Dowell S, Akhter M, Duthuluru S, Huerter C, Hollis BW et al. Ultraviolet-B radiation increases serum 25-hydroxyvitamin D levels: the effect of UVB dose and skin color. J Am Acad Dermatol 2007; 57(4):588-593.

(21) Prentice A. Vitamin D deficiency: a global perspective. Nutr Rev 2008; 66(10 Suppl 2):S153-S164.

(22) Mehta S, Giovannucci E, Mugusi FM, Spiegelman D, Aboud S, Hertzmark E et al. Vitamin D status of HIV-infected women and its association with HIV disease progression, anemia, and mortality. PLoS ONE 2010; 5(1):e8770.

(23) Mehta S, Hunter DJ, Mugusi FM, Spiegelman D, Manji KP, Giovannucci EL et al. Perinatal outcomes, including mother-to-child transmission of HIV, and child mortality and their association with maternal vitamin D status in Tanzania. J Infect Dis 2009; 200(7):1022-1030.

(24) Pettifor JM. Vitamin D &/or calcium deficiency rickets in infants & children: a global perspective. Indian J Med Res 2008; 127(3):245-249.

(25) Villamor E. A potential role for vitamin D on HIV infection? Nutr Rev 2006; 64(5 Pt 1):226-233.

(26) Stephensen CB, Marquis GS, Kruzich LA, Douglas SD, Aldrovandi GM, Wilson CM. Vitamin D status in adolescents and young adults with HIV infection. Am J Clin Nutr 2006; 83(5):1135-1141.

(27) Girasole G, Wang JM, Pedrazzoni M, Pioli G, Balotta C, Passeri M et al. Augmentation of monocyte chemotaxis by 1 alpha,25-dihydroxyvitamin D3. Stimulation of defective migration of AIDS patients. J Immunol 1990; 145(8):2459-2464.

(28) Haug CJ, Muller F, Aukrust P, Froland SS. Different effect of 1,25-dihydroxyvitamin D3 on replication of Mycobacterium avium in monocyte-derived macrophages from human immunodeficiency virus-infected subjects and healthy controls. Immunol Lett 1998; 63(2):107-112.

(29) Teichmann J, Stephan E, Discher T, Lange U, Federlin K, Stracke H et al. Changes in calciotropic hormones and biochemical markers of bone metabolism in patients with human immunodeficiency virus infection. Metabolism 2000; 49(9):1134-1139.

(30) Teichmann J, Stephan E, Lange U, Discher T, Friese G, Lohmeyer J et al. Osteopenia in HIV-infected women prior to highly active antiretroviral therapy. J Infect 2003; 46(4):221-227.

(31) Haug C, Muller F, Aukrust P, Froland SS. Subnormal serum concentration of 1,25-vitamin D in human immunodeficiency virus infection: correlation with degree of immune deficiency and survival. J Infect Dis 1994; 169(4):889-893.

(32) de Luis DA, Bachiller P, Aller R, de Luis J, Izaola O, Terroba MC et al. Relation among micronutrient intakes with CD4 count in HIV infected patients. Nutr Hosp 2002; 17(6):285-289.

(33) Heaney RP. Vitamin D endocrine physiology. J Bone Miner Res 2007; 22(S2):V25-V27.

(34) Aloia JF, Patel M, Dimaano R, Li-Ng M, Talwar SA, Mikhail M et al. Vitamin D intake to attain a desired serum 25-hydroxyvitamin D concentration. Am J Clin Nutr 2008; 87(6):1952-1958.

(35) Vieth R, Chan PC, MacFarlane GD. Efficacy and safety of vitamin D3 intake exceeding the lowest observed adverse effect level. Am J Clin Nutr 2001; 73(2):288-294.

(36) Holick MF. High prevalence of vitamin D inadequacy and implications for health. Mayo Clin Proc 2006; 81(3):353-373.

(37) Holick MF. Vitamin D. In: Shils ME, Shike M, Ross AC, Caballero B, Cousins RJ, editors. Modern Nutrition in Health and Disease. Philadelphia: Lippincott Williams & Wilkins, 2006: 376-395.
