# Supplementary material for: Vitamin D₃ Supplementation in Batswana Children and Adults with HIV: A Pilot Double Blind Randomized Controlled Trial
Source: PLoS One. 2015 Feb 23;10(2):e0117123. doi: 10.1371/journal.pone.0117123 (PMC4338235; doi:10.1371/journal.pone.0117123)
Supplement: S2 Study Dataset — (PDF) [file pone.0117123.s007.pdf]

Botswana Vitamin D Supplementation Dosing Study - Longitudinal Data Set for Manuscript "Vitamin D3 Supplementation in Batswana Children and Adults with HIV: a Pilot Double

ID	Visit	Time wks	Sex	Age yrs	Age Group	HIV Mode of Acquisition	Season	Percent Adherence to vitamin D3 supplement	Vitamin D3 dose IU	CD4 Count mm3	CD4 Count 1=low, 2=high	CD4 %	CD4 % 1=low, 2=high	HIV RNA Viral Load	HIV RNA (log) Viral Load	HIV RNA Detectable (25)
1001	Baseline	0	M	8.30	4-8 yrs	Perinatally Acquired HIV	summer	96	7000	1417	0	33.13	0	<25	1.4	0
1001	Week #6	6	M	8.43	4-8 yrs	Perinatally Acquired HIV	summer	96	7000							
1001	Week #12	12	M	8.61	4-8 yrs	Perinatally Acquired HIV	summer	96	7000	1070	0	32.86	0	<400		
1002	Baseline	0	F	6.69	4-8 yrs	Perinatally Acquired HIV	summer	83	4000	1354	0	36.97	0	<25	1.4	0
1002	Week #6	6	F	6.80	4-8 yrs	Perinatally Acquired HIV	summer	83	4000							
1002	Week #12	12	F	6.91	4-8 yrs	Perinatally Acquired HIV	summer	83	4000	1265	0	34.72	0	42	1.62	1
1003	Baseline	0	M	7.77	4-8 yrs	Perinatally Acquired HIV	fall	66	7000	1252	0	38.95	0	<25	1.4	0
1003	Week #6	6	M	7.88	4-8 yrs	Perinatally Acquired HIV	fall	66	7000							
1003	Week #12	12	M	8.00	4-8 yrs	Perinatally Acquired HIV	fall	66	7000	1093	0	42.85	0	<25	1.4	0
1004	Baseline	0	M	8.13	4-8 yrs	Perinatally Acquired HIV	fall	85	4000	1100	0	39.17	0	200	2.3	1
1004	Week #6	6	M	8.24	4-8 yrs	Perinatally Acquired HIV	fall	85	4000							
1004	Week #12	12	M	8.36	4-8 yrs	Perinatally Acquired HIV	fall	85	4000	857	0	41.49	0	62	1.79	1
1005	Baseline	0	F	8.88	4-8 yrs	Perinatally Acquired HIV	fall	83	4000	766	0	27.29	1	<25	1.4	0
1005	Week #6	6	F	9.00	4-8 yrs	Perinatally Acquired HIV	fall	83	4000							
1005	Week #12	12	F	9.11	4-8 yrs	Perinatally Acquired HIV	fall	83	4000	563	0	30.88	0	<25	1.4	0
1006	Baseline	0	M	7.55	4-8 yrs	Perinatally Acquired HIV	fall	90	7000	1539	0	38.61	0	<400		
1006	Week #6	6	M	7.67	4-8 yrs	Perinatally Acquired HIV	fall	90	7000							
1006	Week #12	12	M	7.85	4-8 yrs	Perinatally Acquired HIV	fall	90	7000	1385	0	39.55	0	<25	1.4	0
1007	Baseline	0	F	7.97	4-8 yrs	Perinatally Acquired HIV	fall	100	4000	779	0	33.33	0	<25	1.4	0
1007	Week #6	6	F	8.08	4-8 yrs	Perinatally Acquired HIV	fall	100	4000							
1007	Week #12	12	F	8.20	4-8 yrs	Perinatally Acquired HIV	fall	100	4000					<400		
1008	Baseline	0	M	6.04	4-8 yrs	Perinatally Acquired HIV	fall	100	7000	1361	0	23.99	1	<25	1.4	0
1008	Week #6	6	M	6.15	4-8 yrs	Perinatally Acquired HIV	fall	100	7000							
1008	Week #12	12	M	6.30	4-8 yrs	Perinatally Acquired HIV	fall	100	7000	1523	0	28.25	1	<400		
1009	Baseline	0	M	7.52	4-8 yrs	Perinatally Acquired HIV	fall	89	4000	1666	0	41.79	0	<25	1.4	0
1009	Week #6	6	M	7.64	4-8 yrs	Perinatally Acquired HIV	fall	89	4000							
1009	Week #12	12	M	7.79	4-8 yrs	Perinatally Acquired HIV	fall	89	4000	1606	0	40.42	0	<25	1.4	0
1010	Baseline	0	F	5.88	4-8 yrs	Perinatally Acquired HIV	fall	100	7000	1662	0	40.56	0	<25	1.4	0
1010	Week #6	6	F	6.00	4-8 yrs	Perinatally Acquired HIV	fall	100	7000							
1010	Week #12	12	F	6.13	4-8 yrs	Perinatally Acquired HIV	fall	100	7000	1322	0	45.2	0	<25	1.4	0
1011	Baseline	0	F	7.43	4-8 yrs	Perinatally Acquired HIV	fall	100	4000	607	0	35.99	0	<25	1.4	0
1011	Week #6	6	F	7.54	4-8 yrs	Perinatally Acquired HIV	fall	100	4000							
1011	Week #12	12	F	7.68	4-8 yrs	Perinatally Acquired HIV	fall	100	4000	845	0	28.31	1	<400		
1012	Baseline	0	F	7.39	4-8 yrs	Perinatally Acquired HIV	fall	76	7000	1657	0	51.48	0	<25	1.4	0
1012	Week #6	6	F	7.50	4-8 yrs	Perinatally Acquired HIV	fall	76	7000							
1012	Week #12	12	F	7.64	4-8 yrs	Perinatally Acquired HIV	fall	76	7000	1712	0	62.31	0	<400		
2001	Baseline	0	F	11.14	9-13 yrs	Perinatally Acquired HIV	summer	98	4000	749	0	49.16	0	<25	1.4	0
2001	Week #6	6	F	11.26	9-13 yrs	Perinatally Acquired HIV	summer	98	4000							
2001	Week #12	12	F	11.37	9-13 yrs	Perinatally Acquired HIV	summer	98	4000							
2002	Baseline	0	F	10.24	9-13 yrs	Perinatally Acquired HIV	summer	81	4000					<25	1.4	0
2002	Week #6	6	F	10.35	9-13 yrs	Perinatally Acquired HIV	summer	81	4000							
2002	Week #12	12	F	10.47	9-13 yrs	Perinatally Acquired HIV	summer	81	4000	511	0	32.73	0	<400		
2003	Baseline	0	M	9.17	9-13 yrs	Perinatally Acquired HIV	summer	85	7000	1516	0	40.08	0	<25	1.4	0
2003	Week #6	6	M	9.29	9-13 yrs	Perinatally Acquired HIV	summer	85	7000							
2003	Week #12	12	M	9.40	9-13 yrs	Perinatally Acquired HIV	summer	85	7000	1088	0	39.77	0	<25	1.4	0
2004	Baseline	0	M	12.96	9-13 yrs	Perinatally Acquired HIV	summer	83	7000	762	0	25.88	1			
2004	Week #6	6	M	13.08	9-13 yrs	Perinatally Acquired HIV	summer	83	7000							

Botswana Vitamin D Supplementation Dosing Study - Longitudinal Data Set for Manuscript "Vitamin D3 Supplementation in Batswana Children and Adults with HIV: a Pilot Double

ID	Visit	Time wks	Sex	Age yrs	Age Group	HIV Mode of Acquisition	Season	Percent Adherence to vitamin D3 supplement	Vitamin D3 dose IU	CD4 Count mm3	CD4 Count 1=low, 2=high	CD4 %	CD4 % 1=low, 2=high	HIV RNA Viral Load	HIV RNA (log) Viral Load	HIV RNA Detectable (25)
2004	Week #12	12	M	13.19	9-13 yrs	Perinatally Acquired HIV	summer	83	7000	505	0	27.33	1	<25	1.4	0
2005	Baseline	0	M	11.33	9-13 yrs		summer	100	4000	1007	0	43.45	0	27	1.43	1
2005	Week #6	6	M	11.45	9-13 yrs		summer	100	4000							
2005	Week #12	12	M	11.57	9-13 yrs		summer	100	4000	1220	0	38.19	0	<25	1.4	0
2006	Baseline	0	M	9.68	9-13 yrs	Perinatally Acquired HIV	summer	80	7000	1234	0	46.53	0	<25	1.4	0
2006	Week #6	6	M	9.80	9-13 yrs	Perinatally Acquired HIV	summer	80	7000							
2006	Week #12	12	M	9.93	9-13 yrs	Perinatally Acquired HIV	summer	80	7000	1184	0	48.29	0	<400		
2007	Baseline	0	M	13.80	9-13 yrs	Perinatally Acquired HIV	summer	36	7000	468	0	30.92	0	120	2.08	1
2007	Week #6	6	M	13.92	9-13 yrs	Perinatally Acquired HIV	summer	36	7000							
2007	Week #12	12	M	14.04	9-13 yrs	Perinatally Acquired HIV	summer	36	7000	426	1	32.29	0	<25	1.4	0
2008	Baseline	0	F	13.24	9-13 yrs	Perinatally Acquired HIV	fall	100	4000	874	0	38.39	0	<25	1.4	0
2008	Week #6	6	F	13.36	9-13 yrs	Perinatally Acquired HIV	fall	100	4000							
2008	Week #12	12	F	13.47	9-13 yrs	Perinatally Acquired HIV	fall	100	4000	953	0	39.63	0	<400		
2009	Baseline	0	M	10.88	9-13 yrs	Perinatally Acquired HIV	fall	73	7000	135	1	9.23	1	780	2.89	1
2009	Week #6	6	M	11.00	9-13 yrs	Perinatally Acquired HIV	fall	73	7000							
2009	Week #12	12	M	11.11	9-13 yrs	Perinatally Acquired HIV	fall	73	7000	122	1	12.13	1	44	1.64	1
2010	Baseline	0	F	11.91	9-13 yrs	Perinatally Acquired HIV	fall	90	4000	991	0	33.82	0	<25	1.4	0
2010	Week #6	6	F	12.03	9-13 yrs	Perinatally Acquired HIV	fall	90	4000							
2010	Week #12	12	F	12.21	9-13 yrs	Perinatally Acquired HIV	fall	90	4000	747	0	35.64	0	<25	1.4	0
2011	Baseline	0	F	13.41	9-13 yrs	Perinatally Acquired HIV	fall	86	7000	667	0	37.33	0	<25	1.4	0
2011	Week #6	6	F	13.54	9-13 yrs	Perinatally Acquired HIV	fall	86	7000							
2011	Week #12	12	F	13.64	9-13 yrs	Perinatally Acquired HIV	fall	86	7000	605	0	37.11	0	<25	1.4	0
2012	Baseline	0	F	9.08	9-13 yrs	Perinatally Acquired HIV	fall	47	4000	2202	2	51.23	0	<25	1.4	0
2012	Week #6	6	F	9.20	9-13 yrs	Perinatally Acquired HIV	fall	47	4000							
2012	Week #12	12	F	9.31	9-13 yrs	Perinatally Acquired HIV	fall	47	4000	1320	0	47.01	0	<25	1.4	0
3001	Baseline	0	M	14.04	14-18 yrs	Perinatally Acquired HIV	fall	85	7000	143	1	16.44	1	110	2.04	1
3001	Week #6	6	M	14.16	14-18 yrs	Perinatally Acquired HIV	fall	85	7000							
3001	Week #12	12	M	14.27	14-18 yrs	Perinatally Acquired HIV	fall	85	7000	359	1	16.19	1	75	1.88	1
3002	Baseline	0	M	15.02	14-18 yrs	Perinatally Acquired HIV	fall	90	4000	699	0	35.42	0	<25	1.4	0
3002	Week #6	6	M	15.13	14-18 yrs	Perinatally Acquired HIV	fall	90	4000							
3002	Week #12	12	M	15.27	14-18 yrs	Perinatally Acquired HIV	fall	90	4000	549	0	37.09	0	<400		
3003	Baseline	0	F	14.86	14-18 yrs	Perinatally Acquired HIV	fall	92	7000	371	1	26.07	1	6100	3.79	1
3003	Week #6	6	F	14.97	14-18 yrs	Perinatally Acquired HIV	fall	92	7000							
3003	Week #12	12	F	15.10	14-18 yrs	Perinatally Acquired HIV	fall	92	7000	272	1	28.4	1	6800	3.83	1
3004	Baseline	0	F	14.74	14-18 yrs	Perinatally Acquired HIV	fall	83	7000	1350	0	39.11	0	<25	1.4	0
3004	Week #6	6	F	14.86	14-18 yrs	Perinatally Acquired HIV	fall	83	7000							
3004	Week #12	12	F	14.97	14-18 yrs	Perinatally Acquired HIV	fall	83	7000	868	0	44.48	0	<25	1.4	0
3005	Baseline	0	M	15.63	14-18 yrs	Perinatally Acquired HIV	fall	83	4000	586	0	34.58	0	<25	1.4	0
3005	Week #6	6	M	15.75	14-18 yrs	Perinatally Acquired HIV	fall	83	4000							
3005	Week #12	12	M	15.86	14-18 yrs	Perinatally Acquired HIV	fall	83	4000	389	1	30.4	0	<25	1.4	0
3006	Baseline	0	F	17.97	14-18 yrs	Perinatally Acquired HIV	fall	88	4000	731	0	31.43	0	<400		
3006	Week #6	6	F	18.09	14-18 yrs	Perinatally Acquired HIV	fall	88	4000							
3006	Week #12	12	F	18.24	14-18 yrs	Perinatally Acquired HIV	fall	88	4000	1001	0	37.66	0	<400		
3007	Baseline	0	F	15.20	14-18 yrs	Perinatally Acquired HIV	fall	92	7000	944	0	35.51	0	<25	1.4	0
3007	Week #6	6	F	15.32	14-18 yrs	Perinatally Acquired HIV	fall	92	7000							
3007	Week #12	12	F	15.43	14-18 yrs	Perinatally Acquired HIV	fall	92	7000	729	0	35.89	0	<25	1.4	0
3008	Baseline	0	F	16.28	14-18 yrs	Perinatally Acquired HIV	fall	93	4000	1021	0	41.53	0	<25	1.4	0

Botswana Vitamin D Supplementation Dosing Study - Longitudinal Data Set for Manuscript "Vitamin D3 Supplementation in Batswana Children and Adults with HIV: a Pilot Double

ID	Visit	Time wks	Sex	Age yrs	Age Group	HIV Mode of Acquisition	Season	Percent Adherence to vitamin D3 supplement	Vitamin D3 dose IU	CD4 Count mm3	CD4 Count 1=low, 2=high	CD4 %	CD4 % 1=low, 2=high	HIV RNA Viral Load	HIV RNA (log) Viral Load	HIV RNA Detectable (25)
3008	Week #6	6	F	16.41	14-18 yrs	Perinatally Acquired HIV	fall	93	4000							
3008	Week #12	12	F	16.52	14-18 yrs	Perinatally Acquired HIV	fall	93	4000	853	0	45.27	0	15000	4.18	1
3009	Baseline	0	M	14.20	14-18 yrs	Perinatally Acquired HIV	fall	86	4000	644	0	35.96	0	<400		
3009	Week #6	6	M	14.32	14-18 yrs	Perinatally Acquired HIV	fall	86	4000							
3009	Week #12	12	M	14.44	14-18 yrs	Perinatally Acquired HIV	fall	86	4000	1011	0	34.71	0	<25	1.4	0
3010	Baseline	0	M	15.47	14-18 yrs	Perinatally Acquired HIV	fall	64	7000	67	1	7.14	1	40	1.6	1
3010	Week #6	6	M	15.58	14-18 yrs	Perinatally Acquired HIV	fall	64	7000							
3010	Week #12	12	M	15.70	14-18 yrs	Perinatally Acquired HIV	fall	64	7000	156	1	8.83	1	<400		
3011	Baseline	0	M	14.99	14-18 yrs	Perinatally Acquired HIV	fall	91	7000	562	0	31.78	0	<25	1.4	0
3011	Week #6	6	M	15.11	14-18 yrs	Perinatally Acquired HIV	fall	91	7000							
3011	Week #12	12	M	15.28	14-18 yrs	Perinatally Acquired HIV	fall	91	7000	628	0	32.84	0	<25	1.4	0
3012	Baseline	0	F	15.32	14-18 yrs	Perinatally Acquired HIV	fall	85	4000	1016	0	30.44	0	<25	1.4	0
3012	Week #6	6	F	15.44	14-18 yrs	Perinatally Acquired HIV	fall	85	4000							
3012	Week #12	12	F	15.55	14-18 yrs	Perinatally Acquired HIV	fall	85	4000	947	0	32.2	0	<25	1.4	0
4001	Baseline	0	F	20.00	19-29 yrs	Perinatally Acquired HIV	summer	82	7000	608	0	36.77	0	<25	1.4	0
4001	Week #6	6	F	20.11	19-29 yrs	Perinatally Acquired HIV	summer	82	7000							
4001	Week #12	12	F	20.23	19-29 yrs	Perinatally Acquired HIV	summer	82	7000	589	0	28.79	1	<25	1.4	0
4002	Baseline	0	F	29.35	19-29 yrs	Behaviorally Acquired	fall	88	4000	596	0	24.61	1	<25	1.4	0
4002	Week #6	6	F	29.46	19-29 yrs	Behaviorally Acquired	fall	88	4000							
4002	Week #12	12	F	29.58	19-29 yrs	Behaviorally Acquired	fall	88	4000	535	0	29.85	1	<25	1.4	0
4003	Baseline	0	F	28.38	19-29 yrs	Behaviorally Acquired	fall	100	7000	195	1	11.27	1	<25	1.4	0
4003	Week #6	6	F	28.49	19-29 yrs	Behaviorally Acquired	fall	100	7000							
4003	Week #12	12	F	28.62	19-29 yrs	Behaviorally Acquired	fall	100	7000	205	1	12.3	1	<25	1.4	0
4004	Baseline	0	F	24.02	19-29 yrs	Behaviorally Acquired	fall	100	4000	267	1	31.59	0	<25	1.4	0
4004	Week #6	6	F	24.14	19-29 yrs	Behaviorally Acquired	fall	100	4000							
4004	Week #12	12	F	24.25	19-29 yrs	Behaviorally Acquired	fall	100	4000	484	1	33.54	0	<25	1.4	0
4005	Baseline	0	F	29.83	19-29 yrs	Behaviorally Acquired	fall	46	7000	526	0	40.59	0	170	2.23	1
4005	Week #6	6	F	29.95	19-29 yrs	Behaviorally Acquired	fall	46	7000							
4005	Week #12	12	F	30.06	19-29 yrs	Behaviorally Acquired	fall	46	7000	455	1	38.72	0	<25	1.4	0
4006	Baseline	0	F	29.33	19-29 yrs	Behaviorally Acquired	fall	83	4000	611	0	19.52	1	37	1.57	1
4006	Week #6	6	F	29.44	19-29 yrs	Behaviorally Acquired	fall	83	4000							
4006	Week #12	12	F	29.56	19-29 yrs	Behaviorally Acquired	fall	83	4000	508	0	23.18	1	<25	1.4	0
4007	Baseline	0	M	29.05	19-29 yrs	Behaviorally Acquired	fall	82	7000	265	1	19.58	1	<25	1.4	0
4007	Week #6	6	M	29.16	19-29 yrs	Behaviorally Acquired	fall	82	7000							
4007	Week #12	12	M	29.28	19-29 yrs	Behaviorally Acquired	fall	82	7000	247	1	22.6	1	<25	1.4	0
4008	Baseline	0	M	19.71	19-29 yrs	Perinatally Acquired HIV	fall	83	4000	646	0	25.63	1	<25	1.4	0
4008	Week #6	6	M	19.83	19-29 yrs	Perinatally Acquired HIV	fall	83	4000							
4008	Week #12	12	M	19.94	19-29 yrs	Perinatally Acquired HIV	fall	83	4000	545	0	26.8	1	<25	1.4	0
4009	Baseline	0	M	19.19	19-29 yrs	Perinatally Acquired HIV	fall	48	7000	491	0	32.26	0	<25	1.4	0
4009	Week #6	6	M	19.30	19-29 yrs	Perinatally Acquired HIV	fall	48	7000							
4009	Week #12	12	M	19.42	19-29 yrs	Perinatally Acquired HIV	fall	48	7000	519	0	39.26	0	<25	1.4	0
4010	Baseline	0	M	20.35	19-29 yrs	Behaviorally Acquired	fall	100	4000	367	1	36.18	0	<25	1.4	0
4010	Week #6	6	M	20.48	19-29 yrs	Behaviorally Acquired	fall	100	4000							
4010	Week #12	12	M	20.58	19-29 yrs	Behaviorally Acquired	fall	100	4000	274	1	29.72	1	<25	1.4	0
4011	Baseline	0	M	19.07	19-29 yrs	Perinatally Acquired HIV	fall	29	4000	909	0	42.71	0	<25	1.4	0
4011	Week #6	6	M	19.20	19-29 yrs	Perinatally Acquired HIV	fall	29	4000							
4011	Week #12	12	M	19.30	19-29 yrs	Perinatally Acquired HIV	fall	29	4000	1040	0	43.52	0	<25	1.4	0

Botswana Vitamin D Supplementation Dosing Study - Longitudinal Data Set for Manuscript "Vitamin D3 Supplementation in Batswana Children and Adults with HIV: a Pilot Double

ID	Visit	Time wks	Sex	Age yrs	Age Group	HIV Mode of Acquisition	Season	Percent Adherence to vitamin D3 supplement	Vitamin D3 dose IU	CD4 Count mm3	CD4 Count 1=low, 2=high	CD4 %	CD4 % 1=low, 2=high	HIV RNA Viral Load	HIV RNA (log) Viral Load	HIV RNA Detectable (25)
4012	Baseline	0	M	19.30	19-29 yrs	Perinatally Acquired HIV	fall	71	7000	140	1	11.19	1			
4012	Week #6	6	M	19.41	19-29 yrs	Perinatally Acquired HIV	fall	71	7000							
4012	Week #12	12	M	19.53	19-29 yrs	Perinatally Acquired HIV	fall	71	7000	198	1	13.01	1			
5001	Baseline	0	F	37.22	30-50 yrs	Behaviorally Acquired	summer	87	4000	968	0	33.18	0	<25	1.4	0
5001	Week #6	6	F	37.34	30-50 yrs	Behaviorally Acquired	summer	87	4000							
5001	Week #12	12	F	37.45	30-50 yrs	Behaviorally Acquired	summer	87	4000	661	0	34.11	0	<25	1.4	0
5002	Baseline	0	F	33.42	30-50 yrs	Behaviorally Acquired	summer	98	7000	528	0	39.94	0	<25	1.4	0
5002	Week #6	6	F	33.53	30-50 yrs	Behaviorally Acquired	summer	98	7000							
5003	Baseline	0	F	32.57	30-50 yrs	Behaviorally Acquired	summer	85	4000	645	0	27	1	<400		
5003	Week #6	6	F	32.69	30-50 yrs	Behaviorally Acquired	summer	85	4000							
5003	Week #12	12	F	32.80	30-50 yrs	Behaviorally Acquired	summer	85	4000	572	0	21.92	1	<25	1.4	0
5004	Baseline	0	F	34.38	30-50 yrs	Behaviorally Acquired	summer	77	4000	756	0	33	0	<400		
5004	Week #6	6	F	34.49	30-50 yrs	Behaviorally Acquired	summer	77	4000							
5004	Week #12	12	F	34.61	30-50 yrs	Behaviorally Acquired	summer	77	4000	540	0	33.29	0	<25	1.4	0
5005	Baseline	0	M	41.70	30-50 yrs	Behaviorally Acquired	summer	76	7000	730	0	22.01	1	<25	1.4	0
5005	Week #6	6	M	41.81	30-50 yrs	Behaviorally Acquired	summer	76	7000							
5005	Week #12	12	M	41.96	30-50 yrs	Behaviorally Acquired	summer	76	7000	930	0	28.27	1	<25	1.4	0
5006	Baseline	0	M	41.88	30-50 yrs	Behaviorally Acquired	summer	100	7000	235	1	17.95	1	<25	1.4	0
5006	Week #6	6	M	41.99	30-50 yrs	Behaviorally Acquired	summer	100	7000							
5006	Week #12	12	M	42.11	30-50 yrs	Behaviorally Acquired	summer	100	7000	204	1	18.65	1	<25	1.4	0
5007	Baseline	0	F	41.43	30-50 yrs	Behaviorally Acquired	summer	87	4000	374	1	19.18	1	<25	1.4	0
5007	Week #6	6	F	41.54	30-50 yrs	Behaviorally Acquired	summer	87	4000							
5007	Week #12	12	F	41.66	30-50 yrs	Behaviorally Acquired	summer	87	4000	483	1	23.11	1	<25	1.4	0
5008	Baseline	0	F	40.54	30-50 yrs	Behaviorally Acquired	summer	86	7000	1244	0	45.81	0	<25	1.4	0
5008	Week #6	6	F	40.66	30-50 yrs	Behaviorally Acquired	summer	86	7000							
5008	Week #12	12	F	40.77	30-50 yrs	Behaviorally Acquired	summer	86	7000	944	0	43.06	0	<25	1.4	0
5009	Baseline	0	M	44.53	30-50 yrs	Behaviorally Acquired	summer	89	4000	483	1	30.21	0	<25	1.4	0
5009	Week #6	6	M	44.65	30-50 yrs	Behaviorally Acquired	summer	89	4000							
5009	Week #12	12	M	44.76	30-50 yrs	Behaviorally Acquired	summer	89	4000	581	0	32.25	0	<25	1.4	0
5010	Baseline	0	M	42.54	30-50 yrs	Behaviorally Acquired	summer	84	7000	187	1	20.57	1	55	1.74	1
5010	Week #6	6	M	42.65	30-50 yrs	Behaviorally Acquired	summer	84	7000							
5010	Week #12	12	M	42.76	30-50 yrs	Behaviorally Acquired	summer	84	7000	333	1	19.77	1	<25	1.4	0
5011	Baseline	0	M	36.87	30-50 yrs	Behaviorally Acquired	summer	100	7000	257	1	29.3	1	<25	1.4	0
5011	Week #6	6	M	37.01	30-50 yrs	Behaviorally Acquired	summer	100	7000							
5011	Week #12	12	M	37.09	30-50 yrs	Behaviorally Acquired	summer	100	7000	422	1	34.26	0	<25	1.4	0
5012	Baseline	0	M	45.93	30-50 yrs	Behaviorally Acquired	summer	89	4000	263	1	16.23	1	<25	1.4	0
5012	Week #6	6	M	46.06	30-50 yrs	Behaviorally Acquired	summer	89	4000							
5012	Week #12	12	M	46.16	30-50 yrs	Behaviorally Acquired	summer	89	4000	305	1	17.49	1	<25	1.4	0

Botswana Vitamin D S Blind Randomized Controlled Trial" (Steenhoff AP et al)

ID	Visit	Time wks	Serum 25(OH)D ng/mL	Serum 25(OH)D category 1=<19, 2=20-31, 3=≥32 ng/mL	Serum PTH pg/mL	Serum 1,25(OH)D pg/mL	Serum Vitamin D Binding Protein umol/L	Free 25(OH)D pg/mL	Bioavailable 25(OH)D ng/mL	Bioavailable/ Total 25(OH)D ratio	Serum Magnesium mmolL	Magnesium 0=in range, 1=low, 2=high	Serum Phosphorous mmolL	Phosphorus 0=in range, 1=low, 2=high
1001	Baseline	0	38.7	3	5.67	33.91	2.09	24.11	8.83	22.81	0.93	0	1.33	0
1001	Week #6	6	38.7	3							1.01	0	1.09	1
1001	Week #12	12	38.5	3	16.14	44.14	1.04	39.38	14.88	38.66	0.92	0	1.39	0
1002	Baseline	0	42.6	3	18.13	51.54					0.96	0	1.55	0
1002	Week #6	6	69.5	3			2.03	46.09	15.94	22.93	0.95	0	1.32	0
1002	Week #12	12	73.1	3	12.51	86.86	1.44	64.41	21.60	29.55	1.39	2	1.58	0
1003	Baseline	0	26.7	2	22.07	41.56	1.69	19.52	6.97	26.11	0.90	0	1.7	0
1003	Week #6	6	80.1	3							1.04	0	1.69	0
1003	Week #12	12	54.7	3	26.41	52.53	1.37	49.08	16.46	30.09	0.87	0	1.55	0
1004	Baseline	0	40.6	3	13.78	40.67	1.61	30.78	11.50	28.31	0.99	0	1.25	0
1004	Week #6	6	55.9	3							0.97	0	1.53	0
1004	Week #12	12	53.3	3	23.65	46.32	1.31	47.92	17.36	32.56	0.90	0	1.37	0
1005	Baseline	0	23.1	2	54.68	54.96					1.03	0	1.7	0
1005	Week #6	6	55.5	3							0.97	0	1.57	0
1005	Week #12	12	51.4	3	23.44	70.36	1.61	39.92	14.23	27.68	0.92	0	1.59	0
1006	Baseline	0	42.4	3	28.50	46.63					0.91	0	1.58	0
1006	Week #6	6	56	3			1.97	38.28	12.23	21.85	0.94	0	1.39	0
1006	Week #12	12	57.6	3	38.29	28.40	1.79	41.96	14.32	24.87	1.00	0	1.81	0
1007	Baseline	0	41.3	3							0.95	0	1.59	0
1007	Week #6	6	60.3	3			1.45	51.60	17.73	29.40	0.99	0	0.91	1
1007	Week #12	12	66.6	3	34.69	54.33	1.62	52.77	18.15	27.24	0.91	0	1.52	0
1008	Baseline	0	48	3	13.07	68.64					1.06	0	1.24	0
1008	Week #6	6	110	3			1.19	116.73	38.39	34.90	0.99	0	1.06	1
1008	Week #12	12	116	3	2.39	85.66	1.15	122.52	44.07	37.99	0.93	0	1.17	1
1009	Baseline	0	55.4	3	17.40	119.39	1.62	44.16	14.41	26.01	0.97	0	1.54	0
1009	Week #6	6	57.2	3							0.89	0	1.31	0
1009	Week #12	12	53.4	3	25.90	62.32	1.22	48.94	19.30	36.15	0.86	0	1.53	0
1010	Baseline	0	25.2	2	39.05	49.65	1.44	20.06	7.93	31.47	1.23	2	1.5	0
1010	Week #6	6	64.4	3							0.96	0	1.4	0
1010	Week #12	12	80.6	3	14.82	76.41	5.10	23.65	8.99	11.15	0.86	0	1.29	0
1011	Baseline	0	39.9	3	20.65	78.78					0.91	0	1.32	0
1011	Week #6	6	68.9	3							0.88	0	1.23	0
1011	Week #12	12	64.9	3	22.81	88.50	4.86	19.86	7.18	11.07	0.82	0	1.55	0
1012	Baseline	0	32.9	3	18.68	78.70					0.86	0	1.39	0
1012	Week #6	6	67.5	3			1.67	52.08	18.08	26.79	1.74	2	1.57	0
1012	Week #12	12	77.8	3	14.58	99.49	1.70	57.62	22.74	29.23	0.88	0	1.37	0
2001	Baseline	0	39.1	3	30.21	167.43	1.59	30.05	11.03	28.20	0.96	0	1.81	2
2001	Week #6	6	54.1	3							0.94	0	1.52	0
2001	Week #12	12	49.8	3	30.58	97.91	1.88							0
2002	Baseline	0	33.6	3	12.72	41.13	1.64	25.58	8.83	26.27	1.04	0	0.84	0
2002	Week #6	6	51.5	3							1.02	0	1.57	0
2002	Week #12	12	57.4	3	22.51	46.48	1.32	52.32	18.28	31.85	0.94	0	1.05	0
2003	Baseline	0	47.4	3	11.76	34.83	2.97	21.88	8.73	18.41	1.08	0	1.56	0
2003	Week #6	6	85.2	3							1.09	0	1.5	0
2003	Week #12	12	96.3	3	1.00	65.98	1.63	77.79	27.38	28.43	0.96	0	1.44	0
2004	Baseline	0	32.5	3	19.20	26.81	2.60	17.24	5.64	17.35	0.84	0	1.52	0
2004	Week #6	6	42.1	3							0.94	0	1.47	0

Botswana Vitamin D S Blind Randomized Controlled Trial" (Steenhoff AP et al)

ID	Visit	Time wks	Serum 25(OH)D ng/mL	Serum 25(OH)D category 1=<19, 2=20-31, 3=≥32 ng/mL	Serum PTH pg/mL	Serum 1,25(OH)D pg/mL	Serum Vitamin D Binding Protein umol/L	Free 25(OH)D pg/mL	Bioavailable 25(OH)D ng/mL	Bioavailable/ Total 25(OH)D ratio	Serum Magnesium mmolL	Magnesium 0=in range, 1=low, 2=high	Serum Phosphorous mmolL	Phosphorus 0=in range, 1=low, 2=high
2004	Week #12	12	40.2	3	14.82	27.98	1.98	26.83	8.87	22.07	0.88	0	1.28	0
2005	Baseline	0	27.1	2	72.39	82.88	1.72	19.79	6.81	25.13	0.99	0	1.6	0
2005	Week #6	6	56	3							0.97	0	1.63	0
2005	Week #12	12	37.5	3	46.57	59.42	2.18	22.95	7.78	20.74	0.90	0	1.29	0
2006	Baseline	0	49.7	3	24.90	54.39	1.71	36.35	13.53	27.22	0.88	0	1.44	0
2006	Week #6	6	94.4	3							1.06	0	1.54	0
2006	Week #12	12	74.4	3	35.00	66.38	1.46	61.48	24.28	32.63	0.93	0	1.46	0
2007	Baseline	0	32.9	3	17.02	79.74	1.57	25.63	9.05	27.51	0.90	0	1.4	0
2007	Week #6	6	40.4	3							1.03	0	1.63	0
2007	Week #12	12	55	3	43.26	105.22	1.50	45.14	16.13	29.33	0.92	0	1.73	0
2008	Baseline	0	33.4	3	75.60	133.63	1.75	23.94	8.50	25.46	0.82	0	1.56	0
2008	Week #6	6	55.3	3							0.89	0	1.37	0
2008	Week #12	12	54.7	3	43.45	181.98	1.95	37.44	12.42	22.71	0.77	0	1.35	0
2009	Baseline	0	30.3	2	16.60	62.81	1.10	31.61	9.84	32.48	0.94	0	1.47	0
2009	Week #6	6	69.8	3							1.06	0	1.95	2
2009	Week #12	12	64.8	3	22.59	56.25	1.84	47.01	15.46	23.86	0.97	0	1.56	0
2010	Baseline	0	32.9	3	31.12	64.84	1.08	34.76	10.98	33.37	0.92	0	1.68	0
2010	Week #6	6	61.3	3							0.94	0	1.81	0
2010	Week #12	12	51.1	3	26.11	83.96	1.42	44.40	14.89	29.14	0.91	0	1.71	0
2011	Baseline	0	27.6	2	43.04	81.28	1.16	26.33	9.47	34.32	0.95	0	1.5	0
2011	Week #6	6	52.6	3							0.98	0	1.56	0
2011	Week #12	12	61.3	3	28.22	129.84	1.12	60.60	23.50	38.33	1.07	0	1.48	0
2012	Baseline	0	48.8	3	25.38	78.53					1.05	0	1.65	0
2012	Week #6	6	75.4	3			1.86	51.88	20.56	27.26	0.92	0	1.38	0
2012	Week #12	12	72.9	3	7.25	92.32	0.96	75.53	33.23	45.59	1.05	0	1.57	0
3001	Baseline	0	15.3	1	76.34	115.31	1.57	12.20	3.71	24.23	0.73	0	1.2	0
3001	Week #6	6	36.2	3							0.90	0	1.71	0
3001	Week #12	12	35.4	3	52.58	135.43	1.02	38.67	12.53	35.39	0.84	0	1.52	0
3002	Baseline	0	38.6	3	27.59	59.03					1.01	0	1.23	0
3002	Week #6	6	54.9	3			1.82	37.77	15.06	27.42	0.94	0	1.16	0
3002	Week #12	12	51.7	3	34.24	88.22	1.36	44.41	16.95	32.78	0.91	0	1.22	0
3003	Baseline	0	19	1	25.35	66.94	1.15	18.97	5.99	31.53	0.96	0	1.38	0
3003	Week #6	6	16.2	1							0.94	0	1.42	0
3003	Week #12	12	16.2	1	43.84	85.09	1.11	15.92	5.60	34.57	0.95	0	1.28	0
3004	Baseline	0	35.2	3	34.44	51.23	1.10	36.66	11.71	33.27	0.88	0	1.31	0
3004	Week #6	6	41.7	3							0.86	0	0.96	0
3004	Week #12	12	48.4	3	20.62	98.06	1.57	38.77	13.25	27.37	0.82	0	1.25	0
3005	Baseline	0	28.2	2	51.87	113.69	1.59	21.93	7.44	26.37	0.93	0	1.43	0
3005	Week #6	6	51.9	3							0.98	0	1.37	0
3005	Week #12	12	53.5	3	20.68	149.30	1.46	43.78	16.70	31.22	0.97	0	1.38	0
3006	Baseline	0	31	2	39.71	56.15	1.48	25.62	8.54	27.53	1.03	0	1.35	0
3006	Week #6	6	50	3							1.02	0	1.21	0
3006	Week #12	12	50.1	3	35.58	61.23	1.45	42.25	14.70	29.35	0.98	0	1.28	0
3007	Baseline	0	32.9	3	59.25	71.08	1.74	24.76	7.40	22.50	0.85	0	1.28	0
3007	Week #6	6	37.4	3							0.72	0	1.23	0
3007	Week #12	12	46.5	3	39.69	83.60	1.55	37.66	12.54	26.96	0.69	0	1.29	0
3008	Baseline	0	26.1	2	37.34	30.81	1.82	18.26	6.18	23.70	1.07	0	1.43	0

Botswana Vitamin D S Blind Randomized Controlled Trial" (Steenhoff AP et al)

ID	Visit	Time wks	Serum 25(OH)D ng/mL	Serum 25(OH)D category 1=<19, 2=20-31, 3=≥32 ng/mL	Serum PTH pg/mL	Serum 1,25(OH)D pg/mL	Serum Vitamin D Binding Protein umol/L	Free 25(OH)D pg/mL	Bioavailable 25(OH)D ng/mL	Bioavailable/ Total 25(OH)D ratio	Serum Magnesium mmolL	Magnesium 0=in range, 1=low, 2=high	Serum Phosphorous mmolL	Phosphorus 0=in range, 1=low, 2=high
3008	Week #6	6	24.8	2							0.72	0	1.23	0
3008	Week #12	12	21.9	2	21.23	46.03	1.53	17.41	5.88	26.86	0.90	0	0.97	0
3009	Baseline	0	38.7	3	16.34	42.49	2.37	22.03	7.57	19.55	0.98	0	1.32	0
3009	Week #6	6	57.3	3							0.90	0	1.15	0
3009	Week #12	12	69.4	3	13.53	18.73	1.41	61.54	20.84	30.03	0.94	0	1.51	0
3010	Baseline	0	24.9	2	46.50	45.51	2.43	14.15	4.13	16.59	0.97	0	1.61	2
3010	Week #6	6	49.3	3							0.93	0	2.04	2
3010	Week #12	12	25.7	2	45.16	36.03					1.04	0	1.88	2
3011	Baseline	0	51.9	3	23.08	46.50					0.86	0	1.33	0
3011	Week #6	6	57.7	3			1.64	44.14	16.18	28.04	0.87	0	1.49	0
3011	Week #12	12	49.2	3	17.85	66.31	1.59	38.45	13.67	27.78	0.76	0	1.54	0
3012	Baseline	0	16.1	1	33.62	44.66	2.55	8.45	2.96	18.38	0.94	0	1.19	0
3012	Week #6	6	28	2							0.91	0	1.22	0
3012	Week #12	12	29.1	2	27.48	59.86					0.91	0	1.14	0
4001	Baseline	0	25.3	2	43.56	53.51	2.14	15.07	5.89	23.29	0.86	0	1.02	0
4001	Week #6	6	44.1	3							0.98	0	0.89	0
4001	Week #12	12	44.2	3	2.50	72.32	1.62	34.30	11.78	26.64	0.90	0	0.89	0
4002	Baseline	0	45.2	3	68.02	30.57	1.60	35.60	12.08	26.72	0.85	0	0.81	0
4002	Week #6	6	56.6	3							0.93	0	0.95	0
4002	Week #12	12	52.8	3	21.41	38.32	1.53	42.91	15.06	28.53	0.81	0	0.93	0
4003	Baseline	0	32.9	3	43.17	36.89	1.50	28.22	7.89	24.00	0.86	0	1.34	0
4003	Week #6	6	33.9	3							0.82	0	1.37	0
4003	Week #12	12	28.9	2	34.44	63.25	1.57	23.46	7.07	24.45	0.71	0	1.35	0
4004	Baseline	0	52.7	3	15.64	55.10	1.45	45.61	14.81	28.11	0.82	0	0.95	0
4004	Week #6	6	62.3	3							0.85	0	0.98	0
4004	Week #12	12	62.5	3	14.51	40.86	2.12	40.45	13.06	20.89	0.79	0	0.98	0
4005	Baseline	0	25.9	2	14.89	43.30	1.52	20.60	7.24	27.97	0.91	0	0.86	0
4005	Week #6	6	35.5	3							0.89	0	0.78	0
4005	Week #12	12	43.1	3	17.28	79.94	1.70	30.63	12.51	29.02	0.87	0	0.65	1
4006	Baseline	0	40.6	3	31.75	85.04	2.31	22.38	9.82	24.19	0.93	0	0.74	0
4006	Week #6	6	62.2	3							0.87	0	0.78	1
4006	Week #12	12	70.8	3	24.27	101.58	1.26	62.09	27.12	38.31	0.96	0	0.72	1
4007	Baseline	0	47.2	3							0.99	0	0.94	0
4007	Week #6	6	63.1	3	16.65	42.86	1.88	43.71	15.91	25.22	0.82	0	1.06	0
4007	Week #12	12	62.8	3	22.59	53.03	1.45	53.82	18.80	29.93	0.95	0	0.81	0
4008	Baseline	0	44.9	3	18.86	79.87	2.31	25.65	9.81	21.86	1.02	0	0.9	0
4008	Week #6	6	55.7	3							0.86	0	1.03	0
4008	Week #12	12	62.4	3	20.70	59.17	1.58	49.02	18.21	29.18	0.85	0	0.93	0
4009	Baseline	0	31.5	2	8.81	76.48	1.34	26.92	10.19	32.36	0.94	0	0.98	0
4009	Week #6	6	45.3	3							0.95	0	0.81	0
4009	Week #12	12	46.8	3	30.30	73.09	1.74	33.06	13.25	28.31	0.85	0	0.85	0
4010	Baseline	0	36.6	3	10.39	52.51	1.41	31.18	10.79	29.49	1.01	0	1.31	0
4010	Week #6	6	46.8	3							0.96	0	1.14	0
4010	Week #12	12	36.5	3	19.22	67.55	1.65	27.13	10.08	27.61	0.92	0	1.17	0
4011	Baseline	0	33.3	3	8.93	76.52	2.02	20.66	8.39	25.21	1.04	0	1.15	0
4011	Week #6	6	29	2							0.94	0	1.37	0
4011	Week #12	12	41.7	3	47.49	82.38	1.63	31.29	11.84	28.39	0.90	0	1.16	0

Botswana Vitamin D S Blind Randomized Controlled Trial" (Steenhoff AP et al)

ID	Visit	Time wks	Serum 25(OH)D ng/mL	Serum 25(OH)D category 1=<19, 2=20-31, 3=≥32 ng/mL	Serum PTH pg/mL	Serum 1,25(OH)D pg/mL	Serum Vitamin D Binding Protein umol/L	Free 25(OH)D pg/mL	Bioavailable 25(OH)D ng/mL	Bioavailable/ Total 25(OH)D ratio	Serum Magnesium mmolL	Magnesium 0=in range, 1=low, 2=high	Serum Phosphorous mmolL	Phosphorus 0=in range, 1=low, 2=high
4012	Baseline	0	28.9	2	8.30	58.38	2.24	16.57	6.62	22.92	1.00	0	0.69	1
4012	Week #6	6	29.1	2							0.89	0	0.75	1
4012	Week #12	12	23.7	2	24.43	46.90	1.51	18.13	7.26	30.64	0.98	0	1.02	0
5001	Baseline	0	32.4	3	40.67	76.64	2.18	18.93	7.69	23.73	0.97	0	1.01	0
5001	Week #6	6	35.7	3							1.07	0	1.02	0
5001	Week #12	12	63.4	3	40.22	91.38	2.09	40.90	13.96	22.02	0.92	0	1.02	0
5002	Baseline	0	34.5	3	17.84	48.44	2.30	19.47	7.70	22.31	0.87	0	1.16	0
5002	Week #6	6	85.7	3	14.30	37.15	1.71	64.02	24.70	28.82	0.86	0	0.95	0
5003	Baseline	0	21.4	2	39.23	30.04	1.24	19.51	6.99	32.67	0.86	0	1.13	0
5003	Week #6	6	48.7	3							0.89	0	1.09	0
5003	Week #12	12	75.7	3	34.85	78.74	2.54	41.41	15.00	19.82	0.88	0	1.01	0
5004	Baseline	0	34.6	3	41.17	33.02	1.23	32.14	11.37	32.85	0.94	0	1.45	0
5004	Week #6	6	45.9	3			4.59	14.82	4.86	10.58	0.81	0	1.27	0
5004	Week #12	12	46.4	3	12.11	43.00					0.85	0	1.27	0
5005	Baseline	0	37.9	3	38.00	47.45	1.53	29.11	11.75	31.00	1.03	0	0.88	0
5005	Week #6	6	59	3							0.86	0	1.16	0
5005	Week #12	12	69.9	3	15.55	65.56	2.87	33.38	13.91	19.90	1.11	2	1.19	0
5006	Baseline	0	53.7	3	49.99	68.41	1.84	37.11	14.14	26.34	0.91	0	1.12	0
5006	Week #6	6	70	3			4.67	22.27	8.07	11.52	0.97	0	1.15	0
5006	Week #12	12	86.6	3	30.02	91.27					1.03	0	1.05	0
5007	Baseline	0	26.3	2	58.31	35.25	1.86	17.79	6.45	24.53	0.87	0	1.38	0
5007	Week #6	6	49.2	3							0.96	0	1.1	0
5007	Week #12	12	47.8	3	17.63	33.63	2.57	25.63	8.82	18.45	0.88	0	1.08	0
5008	Baseline	0	33.8	3	23.64	57.50	1.42	29.04	9.45	27.97	0.86	0	1.09	0
5008	Week #6	6	70.2	3			1.59	57.25	19.04	27.13	0.90	0	1.08	0
5008	Week #12	12	69.7	3	5.67	79.05					0.83	0	1.01	0
5009	Baseline	0	48.5	3	38.09	74.02	2.11	29.98	11.26	23.22	0.95	0	1.33	0
5009	Week #6	6	57.2	3	13.88	68.10					1.00	0	1.18	0
5009	Week #12	12	56.5	3		42.76					0.90	0	1.12	0
5010	Baseline	0	33.2	3	45.03	42.39	2.03	20.82	8.01	24.13	0.88	0	0.98	0
5010	Week #6	6	52.7	3							1.03	0	1.07	0
5010	Week #12	12	61.3	3	22.35	39.92	0.99	67.56	24.15	39.39	0.92	0	1.34	0
5011	Baseline	0	37.4	3	33.85	61.21	1.56	29.24	10.53	28.15	0.89	0	0.91	0
5011	Week #6	6	62.3	3							0.91	0	0.97	0
5011	Week #12	12	63.8	3	23.47	37.46					0.90	0	0.89	0
5012	Baseline	0	43.3	3	29.66	47.82	1.78	30.85	11.12	25.67	0.83	0	0.85	1
5012	Week #6	6	64.4	3			1.50	52.38	19.99	31.04	1.02	0	1	0
5012	Week #12	12	66.2	3	26.57	67.61					0.91	0	1	0

Botswana Vitamin D S

ID	Visit	Time wks	Whole Blood Lead ugdl	Whole Blood Lead 0=in range, 1=low, 2=high	Serum Calcium mmolL	Calcium 0=in range, 1=low, 2=high	Serum Albumin gL	Albumin 0=in range, 1=low, 2=high	Corrected Calcium mmolL	Corrected Calcium 0=in range, 1=low, 2=high	Weight kg	Height cm	BMI	BMI category 1=<18, 2=19-29, 3=≥30	BMI category 1=<18, 2=19-24, 3=≥25	Height-for-age Z Score
1001	Baseline	0	2	0	2.31	0	40.43	0	2.3	0	24.2	123.6	15.84	1	1	-1.03
1001	Week #6	6	2	0	2.44	0	41.82	0	2.4	0	24.4	124.7	15.69	1	1	-0.99
1001	Week #12	12	2	0	2.31	0	41.73	0	2.28	0	25.4	125.53	16.12	1	1	-1.00
1002	Baseline	0	2	0	2.36	0	38.04	0	2.4	0	15.17	105.87	13.53	1	1	-2.72
1002	Week #6	6	2	0	2.52	2	38.18	0	2.56	2	15.2	105.83	13.57	1	1	-2.83
1002	Week #12	12	1	0	2.38	0	37.02	0	2.44	0	16.3	107.17	14.19	1	1	-2.65
1003	Baseline	0	3	0	2.23	0	39.42	0	2.24	0	24.1	118.3	17.22	1	1	-1.49
1003	Week #6	6	3	0	2.31	0	40.57	0	2.3	0	23.6	118.97	16.67	1	1	-1.45
1003	Week #12	12	2	0	2.15	1	37.02	0	2.21	0	23.6	119.2	16.61	1	1	-1.57
1004	Baseline	0	2	0	2.42	0	41.24	0	2.4	0	24.1	129.3	14.42	1	1	0.12
1004	Week #6	6	2	0	2.4	0	40.43	0	2.39	0	23.7	128.87	14.27	1	1	-0.04
1004	Week #12	12	2	0	2.28	0	39.99	0	2.28	0	23.7	129.83	14.06	1	1	-0.04
1005	Baseline	0	3	0	2.34	0	38.26	0	2.37	0	27.8	129.47	16.58	1	1	-0.46
1005	Week #6	6	2	0	2.35	0	41.13	0	2.33	0	27.1	130.9	15.82	1	1	-0.29
1005	Week #12	12	3	0	2.31	0	39.35	0	2.32	0	27.4	131.03	15.96	1	1	-0.40
1006	Baseline	0	1	0	2.4	0	38.09	0	2.44	0	21.2	118.2	15.17	1	1	-1.25
1006	Week #6	6	1	0	2.35	0	35.27	1	2.44	0	21.6	119.5	15.13	1	1	-1.19
1006	Week #12	12	3	0	2.48	0	37.68	0	2.48	0	22.5	120.7	15.44	1	1	-1.14
1007	Baseline	0	2	0	2.3	0	35.56	1	2.39	0	17.6	112.43	13.92	1	1	-2.73
1007	Week #6	6	2	0	2.34	0	37.93	0	2.38	0	18.3	112.66	14.42	1	1	-2.84
1007	Week #12	12	2	0	2.26	0	37.96	0	2.3	0	18.4	113.5	14.28	1	1	-2.75
1008	Baseline	0	2	0	2.29	0	39	0	2.31	0	15.2	106.63	13.37	1	1	-1.78
1008	Week #6	6	1	0	2.31	0	36.3	0	2.38	0	16	107.6	13.82	1	1	-1.68
1008	Week #12	12	1	0	2.31	0	39.71	0	2.33	0	16.7	109.1	14.03	1	1	-1.58
1009	Baseline	0	1	0	2.36	0	36.01	1	2.44	0	32.5	118.83	23.02	2	2	-1.14
1009	Week #6	6	1	0	2.5	0	36.92	1	2.56	0	34.4	119.47	24.10	2	2	-1.11
1009	Week #12	12	2	0	2.52	0	43.56	0	2.45	0	34.9	120.17	24.17	2	2	-1.15
1010	Baseline	0	4	0	2.5	0	43.67	0	2.43	0	15.4	104.5	14.10	1	1	-1.92
1010	Week #6	6	3	0	2.38	0	41.05	0	2.36	0	16.3	107.37	14.14	1	1	-1.54
1010	Week #12	12	3	0	2.35	0	41.97	0	2.31	0	16.8	108.2	14.35	1	1	-1.48
1011	Baseline	0	2	0	2.34	0	40.48	0	2.33	0	23.4	126.1	14.72	1	1	0.30
1011	Week #6	6	1	0	2.26	0	35.94	1	2.34	0	23.5	126.97	14.58	1	1	0.36
1011	Week #12	12	1	0	2.35	0	39.93	0	2.35	0	24.5	128.93	14.74	1	1	0.52
1012	Baseline	0	6	0	2.29	0	37.88	0	2.33	0	24.8	122.43	16.55	1	1	-0.26
1012	Week #6	6	4	0	2.21	0	38.33	0	2.24	0	23.67	122.8	15.70	1	1	-0.37
1012	Week #12	12	7	0	2.36	0	43.58	0	2.29	0	25.7	123.87	16.75	1	1	-0.27
2001	Baseline	0	2	0	2.28	0	40.51	0	2.27	0	28.67	141.57	14.30	1	1	-0.45
2001	Week #6	6	2	0	2.22	0	38.48	0	2.25	0	30.8	142.33	15.20	1	1	-0.50
2001	Week #12	12									30.8	142.33	15.20	1	1	-0.58
2002	Baseline	0	4	0	2.39	0	38.1	0	2.43	0	20	121.13	13.63	1	1	-2.76
2002	Week #6	6	4	0	2.47	0	38.22	0	2.51	2	20.8	122.43	13.88	1	1	-2.65
2002	Week #12	12	4	0	2.43	0	38.58	0	2.46	0	20.4	122.87	13.51	1	1	-2.63
2003	Baseline	0	1	0	2.47	0	44.04	0	2.39	0	22.7	122.23	15.19	1	1	-2.04
2003	Week #6	6	1	0	2.53	2	41.14	0	2.51	2	22.6	123.03	14.93	1	1	-1.96
2003	Week #12	12	1	0	2.36	0	38.86	0	2.38	0	23.2	123.5	15.21	1	1	-1.94
2004	Baseline	0	2	0	2.25	0	36.12	1	2.33	0	34.1	146.1	15.98	1	1	-1.24
2004	Week #6	6	2	0	2.36	0	36.7	1	2.43	0	33.83	146.83	15.69	1	1	-1.22

Botswana Vitamin D S

ID	Visit	Time wks	Whole Blood Lead ugdl	Whole Blood Lead 0=in range, 1=low, 2=high	Serum Calcium mmolL	Calcium 0=in range, 1=low, 2=high	Serum Albumin gL	Albumin 0=in range, 1=low, 2=high	Corrected Calcium mmolL	Corrected Calcium 0=in range, 1=low, 2=high	Weight kg	Height cm	BMI	BMI category 1=<18, 2=19-29, 3=≥30	BMI category 1=<18, 2=19-24, 3=≥25	Height-for-age Z Score
2004	Week #12	12	2	0	2.33	0	36.5	0	2.4	0	34	147.53	15.62	1	1	-1.29
2005	Baseline	0	2	0	2.23	0	37.98	0	2.27	0	22.8	128.3	13.85	1	1	-2.42
2005	Week #6	6	2	0	2.38	0	37.95	0	2.42	0	22.7	128.77	13.69	1	1	-2.46
2005	Week #12	12	2	0	2.26	0	37.42	0	2.31	0	23.7	129.97	14.03	1	1	-2.34
2006	Baseline	0	2	0	2.22	0	41.09	0	2.2	0	25.1	128.27	15.26	1	1	-1.40
2006	Week #6	6	1	0	2.41	0	43.54	0	2.34	0	25.1	129.07	15.07	1	1	-1.33
2006	Week #12	12	1	0	2.24	0	43.61	0	2.17	0	25.7	129.63	15.29	1	1	-1.36
2007	Baseline	0	2	0	2.4	0	38.99	0	2.42	0	30.97	140.07	15.79	1	1	-2.68
2007	Week #6	6	1	0	2.42	0	37.96	0	2.46	0	30.9	140.63	15.62	1	1	-2.73
2007	Week #12	12	2	0	2.36	0	39.46	0	2.37	0	32.4	141.53	16.18	1	1	-2.68
2008	Baseline	0	2	0	2.23	0	39.21	0	2.25	0	39.4	157.07	15.97	1	1	-0.14
2008	Week #6	6	2	0	2.32	0	38.42	0	2.35	0	41	157.83	16.46	1	1	-0.12
2008	Week #12	12	2	0	2.19	0	36.62	0	2.26	0	42.6	158.47	16.96	1	1	-0.07
2009	Baseline	0	1	0	2.29	0	34.37	1	2.4	0	26.4	130.9	15.41	1	1	-1.76
2009	Week #6	6	2	0	2.33	0	36.16	1	2.41	0	25.3	131.5	14.63	1	1	-1.72
2009	Week #12	12	2	0	2.26	0	36.31	0	2.33	0	26.3	131.63	15.18	1	1	-1.81
2010	Baseline	0	4	0	2.19	1	34.86	1	2.29	0	28.43	135.5	15.48	1	1	-1.97
2010	Week #6	6	3	0	2.14	1	33.59	1	2.27	0	28.3	135	15.53	1	1	-2.20
2010	Week #12	12	5	0	2.35	0	37.02	0	2.41	0	31.1	136.5	16.69	1	1	-2.17
2011	Baseline	0	1	0	2.32	0	39.72	0	2.33	0	40.5	151.57	17.63	1	1	-1.05
2011	Week #6	6	1	0	2.21	0	38.56	0	2.24	0	41.8	151.93	18.11	2	2	-1.08
2011	Week #12	12	2	0	2.37	0	42.82	0	2.31	0	42.3	152.9	18.09	2	2	-0.98
2012	Baseline	0	2	0	2.46	0	47.79	0	2.3	0	23.9	123.87	15.58	1	1	-1.53
2012	Week #6	6	1	0	2.43	0	43.76	0	2.35	0	24.3	124.03	15.80	1	1	-1.63
2012	Week #12	12	1	0	2.49	0	48.61	0	2.32	0	24.9	124.9	15.96	1	1	-1.54
3001	Baseline	0	2	0	2.09	1	33.54	1	2.22	0	46.07	158.4	18.36	2	2	-0.71
3001	Week #6	6	2	0	2.14	0	31.18	1	2.32	0	47.7	158.43	19.00	2	2	-0.77
3001	Week #12	12	2	0	2.12	0	35.76	0	2.21	0	48.6	159.97	18.99	2	2	-0.73
3002	Baseline	0	2	0	2.35	0	41.9	0	2.31	0	33.9	153.1	14.46	1	1	-2.05
3002	Week #6	6	1	0	2.49	0	44.02	0	2.41	0	33.6	154.3	14.11	1	1	-1.96
3002	Week #12	12	2	0	2.35	0	42.14	0	2.31	0	35.2	155	14.65	1	1	-1.98
3003	Baseline	0	2	0	2.22	0	34.85	1	2.32	0	46.1	163.53	17.24	1	1	0.28
3003	Week #6	6	1	0	2.24	0	34.86	1	2.34	0	46.2	164.63	17.05	1	1	0.43
3003	Week #12	12	1	0	2.28	0	38.84	0	2.3	0	48.8	164.4	18.06	2	2	0.37
3004	Baseline	0	1	0	2.35	0	35.26	1	2.44	0	35.2	148.67	15.93	1	1	-1.99
3004	Week #6	6	1	0	2.34	0	33.53	1	2.47	0	36.9	149.57	16.49	1	1	-1.88
3004	Week #12	12	1	0	2.45	0	37.72	0	2.5	0	37.2	150.03	16.53	1	1	-1.82
3005	Baseline	0	2	0	2.14	1	37.44	0	2.19	1	43.2	158.3	17.24	1	1	-1.77
3005	Week #6	6	2	0	2.24	0	37.81	0	2.28	0	42.8	158.73	16.99	1	1	-1.75
3005	Week #12	12	2	0	2.37	0	42.12	0	2.33	0	43.7	159.4	17.20	1	1	-1.75
3006	Baseline	0	2	0	2.34	0	36.77	1	2.4	0	47.6	149.9	21.18	2	2	-2.04
3006	Week #6	6	2	0	2.3	0	38.31	0	2.33	0	48.2	150.13	21.39	2	2	-2.01
3006	Week #12	12	2	0	2.31	0	38.42	0	2.34	0	48.1	150.83	21.14	2	2	-1.90
3007	Baseline	0	3	0	2.19	0	32.98	1	2.33	0	38.77	150.67	17.08	1	1	-1.76
3007	Week #6	6	1	0	2.2	0	32.78	1	2.34	0	39.7	152.03	17.18	1	1	-1.56
3007	Week #12	12	2	0	2.39	0	36.75	0	2.46	0	39.77	151.37	17.36	1	1	-1.68
3008	Baseline	0	1	0	2.45	0	37.39	0	2.5	0	62.6	167.5	22.31	2	2	0.74

Botswana Vitamin D S

ID	Visit	Time wks	Whole Blood Lead ugdl	Whole Blood Lead 0=in range, 1=low, 2=high	Serum Calcium mmolL	Calcium 0=in range, 1=low, 2=high	Serum Albumin gL	Albumin 0=in range, 1=low, 2=high	Corrected Calcium mmolL	Corrected Calcium 0=in range, 1=low, 2=high	Weight kg	Height cm	BMI	BMI category 1=<18, 2=19-29, 3=≥30	BMI category 1=<18, 2=19-24, 3=≥25	Height-for-age Z Score
3008	Week #6	6	1	0	2.2	0	32.78	1	2.34	0	64.7	167.7	23.01	2	2	0.77
3008	Week #12	12	3	0	2.33	0	37.29	0	2.38	0	65.4	166.83	23.50	2	2	0.63
3009	Baseline	0	2	0	2.36	0	37.92	0	2.4	0	34.8	141.6	17.36	1	1	-2.79
3009	Week #6	6	2	0	2.21	0	39.67	0	2.22	0	33.8	142.6	16.62	1	1	-2.73
3009	Week #12	12	3	0	2.34	0	37.39	0	2.39	0	36.5	143.03	17.84	1	1	-2.79
3010	Baseline	0	2	0	2.1	1	32.22	1	2.26	0	25.9	133.6	14.51	1	1	-4.32
3010	Week #6	6	3	0	2.34	0	45.04	0	2.24	0	25.4	133.43	14.27	1	1	-4.38
3010	Week #12	12	2	0	2.27	0	37.73	0	2.32	0	27.1	134.1	15.07	1	1	-4.42
3011	Baseline	0	2	0	2.34	0	38.19	0	2.38	0	42.3	170.17	14.61	1	1	0.05
3011	Week #6	6	2	0	2.35	0	40.47	0	2.34	0	43.1	171.23	14.70	1	1	0.09
3011	Week #12	12	2	0	2.36	0	39.24	0	2.38	0	46.1	172.47	15.50	1	1	0.16
3012	Baseline	0	1	0	2.24	0	38.65	0	2.27	0	45.6	149.33	20.45	2	2	-1.98
3012	Week #6	6	1	0	2.29	0	40.33	0	2.29	0	45.5	150.1	20.20	2	2	-1.88
3012	Week #12	12	1	0	2.35	0	42.33	0	2.31	0	45.4	149.2	20.39	2	2	-2.03
4001	Baseline	0	1	0	2.35	0	43.18	0	2.29	0	50.77	157.07	20.58	2	2	
4001	Week #6	6	1	0	2.3	0	39.55	0	2.31	0	50.67	156.47	20.70	2	2	
4001	Week #12	12	1	0	2.26	0	37.9	0	2.3	0	54.3	157.07	22.01	2	2	
4002	Baseline	0	1	0	2.17	1	37.45	0	2.22	0	59	153.57	25.02	2	3	
4002	Week #6	6	1	0	2.37	0	40.29	0	2.36	0	60	153.57	25.44	2	3	
4002	Week #12	12	2	0	2.23	0	38.76	0	2.25	0	63.1	153.27	26.86	2	3	
4003	Baseline	0	6	0	2.14	1	30.86	1	2.32	0	68.7	154.94	28.62	2	3	
4003	Week #6	6	9	0	2.19	1	32.92	1	2.33	0	68.1	153.33	28.97	2	3	
4003	Week #12	12	13	2	2.18	1	33.24	1	2.32	0	69.9	154.67	29.22	2	3	
4004	Baseline	0	1	0	2.24	0	35.85	0	2.32	0	74.33	165	27.30	2	3	
4004	Week #6	6	1	0	2.21	0	35.2	0	2.31	0	76.9	164.6	28.38	2	3	
4004	Week #12	12	1	0	2.2	0	35.63	0	2.29	0	78.6	164.4	29.08	2	3	
4005	Baseline	0	1	0	2.27	0	38.81	0	2.29	0	51.9	161.37	19.93	2	2	
4005	Week #6	6	1	0	2.23	0	39.86	0	2.23	0	54.33	160.73	21.03	2	2	
4005	Week #12	12	1	0	2.31	0	45.1	0	2.21	0	52.27	161.1	20.14	2	2	
4006	Baseline	0	1	0	2.48	0	48.49	0	2.31	0	37.7	145.23	17.87	1	1	
4006	Week #6	6	1	0	2.4	0	49.84	0	2.2	0	37.77	145.13	17.93	1	1	
4006	Week #12	12	3	0	2.48	0	48.26	0	2.31	0	38.9	145	18.50	2	2	
4007	Baseline	0	2	0	2.32	0	39.46	0	2.33	0	61.1	170.87	20.93	2	2	
4007	Week #6	6	3	0	2.31	0	40.2	0	2.31	0	62	171	21.20	2	2	
4007	Week #12	12	2	0	2.43	0	38.56	0	2.46	0	61.97	170.53	21.31	2	2	
4008	Baseline	0	3	0	2.45	0	42.25	0	2.41	0	40.33	164.63	14.88	1	1	-1.69
4008	Week #6	6	3	0	2.34	0	43.57	0	2.27	0	40.4	164.13	15.00	1	1	-1.77
4008	Week #12	12	2	0	2.41	0	41.02	0	2.39	0	41.3	163.93	15.37	1	1	-1.80
4009	Baseline	0	1	0	2.47	0	41.81	0	2.43	0	49.3	170.57	16.95	1	1	-0.85
4009	Week #6	6	2	0	2.45	0	44.62	0	2.36	0	49.7	170.7	17.06	1	1	-0.84
4009	Week #12	12	2	0	2.44	0	44.26	0	2.35	0	49	170.5	16.86	1	1	-0.87
4010	Baseline	0	7	0	2.3	0	38.22	0	2.34	0	57.2	172.17	19.30	2	2	
4010	Week #6	6	8	0	2.33	0	38.29	0	2.36	0	55.1	173.13	18.38	2	2	
4010	Week #12	12	13	2	2.28	0	41.02	0	2.26	0	56.77	173.33	18.90	2	2	
4011	Baseline	0	1	0	2.48	0	44.88	0	2.38	0	59.1	175.57	19.17	2	2	-0.15
4011	Week #6	6	2	0	2.34	0	41.83	0	2.3	0	59	176.03	19.04	2	2	-0.09
4011	Week #12	12	1	0	2.3	0	41.78	0	2.26	0	59.5	175.9	19.23	2	2	-0.11

Botswana Vitamin D S

ID	Visit	Time wks	Whole Blood Lead ugdl	Whole Blood Lead 0=in range, 1=low, 2=high	Serum Calcium mmolL	Calcium 0=in range, 1=low, 2=high	Serum Albumin gL	Albumin 0=in range, 1=low, 2=high	Corrected Calcium mmolL	Corrected Calcium 0=in range, 1=low, 2=high	Weight kg	Height cm	BMI	BMI category 1=<18, 2=19-29, 3=≥30	BMI category 1=<18, 2=19-24, 3=≥25	Height-for-age Z Score
4012	Baseline	0	1	0	2.41	0	44.13	0	2.33	0	45.6	159.5	17.92	1	1	-2.39
4012	Week #6	6	1	0	2.38	0	42.73	0	2.33	0	45.2	160.23	17.61	1	1	-2.29
4012	Week #12	12	1	0	2.48	0	44.22	0	2.4	0	44.1	160.33	17.16	1	1	-2.28
5001	Baseline	0	4	0	2.34	0	44.86	0	2.24	0	35.87	157.94	14.38	1	1	
5001	Week #6	6	7	0	2.39	0	40.85	0	2.37	0	34.97	156.43	14.29	1	1	
5001	Week #12	12	8	0	2.17	1	37.69	0	2.22	0	35.9	158.1	14.36	1	1	
5002	Baseline	0	4	0	2.36	0	43.65	0	2.29	0	56.6	159	22.39	2	2	
5002	Week #6	6	3	0	2.27	0	42.6	0	2.22	0	55.73	158.23	22.26	2	2	
5003	Baseline	0	2	0	2.28	0	39.57	0	2.29	0	100.6	154.86	41.95	3	3	
5003	Week #6	6	2	0	2.34	0	41.93	0	2.3	0	99.13	154.8	41.37	3	3	
5003	Week #12	12	2	0	2.27	0	40	0	2.27	0	94.43	152.23	40.75	3	3	
5004	Baseline	0	1	0	2.27	0	39.05	0	2.29	0	52.37	156.3	21.44	2	2	
5004	Week #6	6	1	0	2.21	0	36.18	0	2.29	0	52.67	156.67	21.46	2	2	
5004	Week #12	12	1	0	2.24	0	36.89	0	2.3	0	54.47	157.13	22.06	2	2	
5005	Baseline	0	14	2	2.32	0	44.57	0	2.23	0	59.07	172.9	19.76	2	2	
5005	Week #6	6	9	0	2.3	0	41.31	0	2.27	0	59.5	172.37	20.03	2	2	
5005	Week #12	12	4	0	2.34	0	46.03	0	2.22	0	57.23	172.4	19.26	2	2	
5006	Baseline	0	1	0	2.35	0	42.08	0	2.31	0	42.83	161.4	16.44	1	1	
5006	Week #6	6	1	0	2.37	0	39.99	0	2.37	0	45.57	160.67	17.65	1	1	
5006	Week #12	12	1	0	2.28	0	38.97	0	2.3	0	45.1	160.6	17.49	1	1	
5007	Baseline	0	4	0	2.23	0	40.05	0	2.23	0	58.97	163.27	22.12	2	2	
5007	Week #6	6	3	0	2.4	0	40.01	0	2.4	0	59.5	161.6	22.78	2	2	
5007	Week #12	12	3	0	2.25	0	37.99	0	2.29	0	58.3	161.73	22.29	2	2	
5008	Baseline	0	2	0	2.27	0	35.93	0	2.35	0	53.8	154.63	22.50	2	2	
5008	Week #6	6	1	0	2.27	0	36.72	0	2.34	0	51.47	154.56	21.55	2	2	
5008	Week #12	12	2	0	2.14	1	31.5	1	2.31	0	52.4	154.9	21.84	2	2	
5009	Baseline	0	2	0	2.37	0	41.47	0	2.34	0	61.9	183.37	18.41	2	2	
5009	Week #6	6	9	0	2.29	0	36.42	0	2.36	0	60.13	182.13	18.13	2	2	
5009	Week #12	12	6	0	2.3	0	37.35	0	2.35	0	62.63	181.77	18.96	2	2	
5010	Baseline	0	1	0	2.29	0	42.49	0	2.24	0	55.77	176.07	17.99	1	1	
5010	Week #6	6	3	0	2.38	0	40.02	0	2.38	0	54.5	176.27	17.54	1	1	
5010	Week #12	12	3	0	2.3	0	39.46	0	2.31	0	54.7	175.53	17.75	1	1	
5011	Baseline	0	6	0	2.12	1	39.76	0	2.12	1	71	172.33	23.91	2	2	
5011	Week #6	6	29	2	2.29	0	40.17	0	2.29	0	70.9	172.67	23.78	2	2	
5011	Week #12	12	14	2	2.23	0	40.7	0	2.22	0	71.7	172.03	24.23	2	2	
5012	Baseline	0			2.14	1	39.79	0	2.14	1	56.43	168.3	19.92	2	2	
5012	Week #6	6	1	0	2.38	0	42.14	0	2.34	0	55.77	168.2	19.71	2	2	
5012	Week #12	12	1	0	2.3	0	41.07	0	2.28	0	57.37	168.53	20.20	2	2	

Botswana Vitamin D S

ID	Visit	Time wks	Weight-for-age Z Score	BMI-for-age Z Score	Height-for-age Z < -2.0 0=no, 1=yes	Weight-for-age Z < -2.0 0=no, 1=yes	BMI-for-age Z < -2.0 0=no, 1=yes	BMI category 0=<85th, 1=≥85th percentile	Years since HIV diagnosis	Years on HAART	CDC HIV Classification	CD4 count Category: worst
1001	Baseline	0	-0.60	-0.02	0	0	0	0	7.67	6.55	A (mildly symp)	Cell count >= 500, no evidence of immune suppression
1001	Week #6	6	-0.66	-0.15	0	0	0	0	7.80	6.68		
1001	Week #12	12	-0.50	0.07	0	0	0	0	7.98	6.86	A (mildly symp)	Cell count >= 500, no evidence of immune suppression
1002	Baseline	0	-3.06	-1.52	1	1	0	0	4.51	4.51	C (severely symp)	Cell count >= 500, no evidence of immune suppression
1002	Week #6	6	-3.12	-1.48	1	1	0	0	4.63	4.63		
1002	Week #12	12	-2.50	-0.90	1	1	0	0	4.74	4.74	C (severely symp)	Cell count >= 500, no evidence of immune suppression
1003	Baseline	0	-0.27	0.80	0	0	0	0	7.73	1.60	C (severely symp)	Cell count >= 500, no evidence of immune suppression
1003	Week #6	6	-0.47	0.53	0	0	0	0	7.84	1.72		
1003	Week #12	12	-0.59	0.46	0	0	0	0	7.96	1.84	C (severely symp)	Cell count >= 500, no evidence of immune suppression
1004	Baseline	0	-0.50	-1.04	0	0	0	0	8.13	7.61	N (not symp)	
1004	Week #6	6	-0.69	-1.19	0	0	0	0	8.24	7.72		
1004	Week #12	12	-0.81	-1.42	0	0	0	0	8.36	7.84	N (not symp)	Cell count >= 500, no evidence of immune suppression
1005	Baseline	0	-0.15	0.17	0	0	0	0	1.39	1.34	C (severely symp)	Cell count < 200, severe suppression
1005	Week #6	6	-0.35	-0.23	0	0	0	0	1.50	1.46		
1005	Week #12	12	-0.40	-0.19	0	0	0	0	1.62	1.57	C (severely symp)	Cell count < 200, severe suppression
1006	Baseline	0	-1.03	-0.32	0	0	0	0	4.36	4.30	A (mildly symp)	Cell count >= 500, no evidence of immune suppression
1006	Week #6	6	-1.01	-0.39	0	0	0	0	4.49	4.42		
1006	Week #12	12	-0.82	-0.19	0	0	0	0	4.67	4.61	A (mildly symp)	Cell count >= 500, no evidence of immune suppression
1007	Baseline	0	-2.70	-1.29	1	1	0	0	4.64	4.25	N (not symp)	Cell count >= 500, no evidence of immune suppression
1007	Week #6	6	-2.49	-0.92	1	1	0	0	4.76	4.36		
1007	Week #12	12	-2.51	-1.04	1	1	0	0	4.88	4.48	N (not symp)	Cell count >= 500, no evidence of immune suppression
1008	Baseline	0	-2.68	-2.14	0	1	1	0	3.38	3.30	N (not symp)	Cell count < 200, severe suppression
1008	Week #6	6	-2.25	-1.54	0	1	0	0	3.50	3.41		
1008	Week #12	12	-2.01	-1.28	0	1	0	0	3.64	3.56	N (not symp)	Cell count < 200, severe suppression
1009	Baseline	0	1.57	2.26	0	0	0	1	3.64	3.64	C (severely symp)	Cell count 200-499, moderate suppression
1009	Week #6	6	1.77	2.37	0	0	0	1	3.76	3.76		
1009	Week #12	12	1.74	2.34	0	0	0	1	3.91	3.91	C (severely symp)	Cell count 200-499, moderate suppression
1010	Baseline	0	-2.08	-0.92	0	1	0	0	5.65	5.65	N (not symp)	Cell count >= 500, no evidence of immune suppression
1010	Week #6	6	-1.72	-0.88	0	0	0	0	5.77	5.77		
1010	Week #12	12	-1.53	-0.69	0	0	0	0	5.90	5.90	N (not symp)	Cell count >= 500, no evidence of immune suppression
1011	Baseline	0	-0.16	-0.57	0	0	0	0	1.65	1.65	N (not symp)	Cell count >= 500, no evidence of immune suppression
1011	Week #6	6	-0.20	-0.69	0	0	0	0	1.76	1.76		
1011	Week #12	12	-0.06	-0.60	0	0	0	0	1.90	1.90	N (not symp)	Cell count >= 500, no evidence of immune suppression
1012	Baseline	0	0.25	0.51	0	0	0	0	7.37	7.37	N (not symp)	Cell count >= 500, no evidence of immune suppression
1012	Week #6	6	-0.15	0.04	0	0	0	0	7.48	7.48		
1012	Week #12	12	0.28	0.55	0	0	0	0	7.62	7.62	N (not symp)	Cell count >= 500, no evidence of immune suppression
2001	Baseline	0	-1.52	-1.74	0	0	0	0	7.55	7.55	N (not symp)	Cell count 200-499, moderate suppression
2001	Week #6	6	-1.20	-1.19	0	0	0	0	7.68	7.68		
2001	Week #12	12	-1.26	-1.21	0	0	0	0	7.79	7.79	N (not symp)	Cell count 200-499, moderate suppression
2002	Baseline	0	-3.33	-2.02	1	1	1	0	4.09	4.01		
2002	Week #6	6	-3.15	-1.86	1	1	0	0	4.21	4.12		
2002	Week #12	12	-3.37	-2.18	1	1	1	0	4.32	4.24	N (not symp)	Cell count >= 500, no evidence of immune suppression
2003	Baseline	0	-1.78	-0.65	1	0	0	0	7.56	7.35	N (not symp)	Cell count >= 500, no evidence of immune suppression
2003	Week #6	6	-1.88	-0.86	0	0	0	0	7.67	7.47		
2003	Week #12	12	-1.72	-0.68	0	0	0	0	7.79	7.58	N (not symp)	Cell count >= 500, no evidence of immune suppression
2004	Baseline	0	-1.61	-1.26	0	0	0	0	4.56	4.56		Cell count 200-499, moderate suppression
2004	Week #6	6	-1.72	-1.48	0	0	0	0	4.67	4.67		

Botswana Vitamin D S

ID	Visit	Time wks	Weight-for-age Z Score	BMI-for-age Z Score	Height-for-age Z < -2.0 0=no, 1=yes	Weight-for-age Z < -2.0 0=no, 1=yes	BMI-for-age Z < -2.0 0=no, 1=yes	BMI category 0=<85th, 1=≥85th percentile	Years since HIV diagnosis	Years on HAART	CDC HIV Classification	CD4 count Category: worst
2004	Week #12	12	-1.81	-1.59	0	0	0	0	4.79	4.79	A (mildly symp)	Cell count 200-499, moderate suppression
2005	Baseline	0	-3.25	-2.38	1	1	1	0	8.57	1.57	C (severely symp)	Cell count 200-499, moderate suppression
2005	Week #6	6	-3.41	-2.60	1	1	1	0	8.69	1.69		
2005	Week #12	12	-3.11	-2.29	1	1	1	0	8.81	1.81	C (severely symp)	Cell count 200-499, moderate suppression
2006	Baseline	0	-1.36	-0.73	0	0	0	0	8.58	8.58	C (severely symp)	Cell count >= 500, no evidence of immune suppression
2006	Week #6	6	-1.42	-0.89	0	0	0	0	8.70	8.70		
2006	Week #12	12	-1.36	-0.77	0	0	0	0	8.83	8.83	C (severely symp)	Cell count >= 500, no evidence of immune suppression
2007	Baseline	0	-2.89	-1.69	1	1	0	0		0.97	C (severely symp)	Cell count 200-499, moderate suppression
2007	Week #6	6	-3.05	-1.87	1	1	0	0		1.09		
2007	Week #12	12	-2.79	-1.52	1	1	0	0		1.20	C (severely symp)	Cell count 200-499, moderate suppression
2008	Baseline	0	-0.93	-1.29	0	0	0	0	10.55	8.89	C (severely symp)	Cell count < 200, severe suppression
2008	Week #6	6	-0.78	-1.07	0	0	0	0	10.67	9.00		
2008	Week #12	12	-0.60	-0.84	0	0	0	0	10.78	9.12	C (severely symp)	Cell count < 200, severe suppression
2009	Baseline	0	-1.82	-0.96	0	0	0	0	0.23	0.15	B (moderately symp)	Cell count < 200, severe suppression
2009	Week #6	6	-2.19	-1.56	0	1	0	0	0.34	0.26		
2009	Week #12	12	-2.02	-1.19	0	1	0	0	0.46	0.38	B (moderately symp)	Cell count < 200, severe suppression
2010	Baseline	0	-2.11	-1.18	0	1	0	0		0.74	C (severely symp)	Cell count 200-499, moderate suppression
2010	Week #6	6	-2.27	-1.21	1	1	0	0		0.86		
2010	Week #12	12	-1.77	-0.64	1	0	0	0		1.03	C (severely symp)	Cell count 200-499, moderate suppression
2011	Baseline	0	-0.85	-0.51	0	0	0	0	5.61	5.48	A (mildly symp)	Cell count >= 500, no evidence of immune suppression
2011	Week #6	6	-0.75	-0.35	0	0	0	0	5.74	5.61		
2011	Week #12	12	-0.72	-0.38	0	0	0	0	5.84	5.71	A (mildly symp)	Cell count >= 500, no evidence of immune suppression
2012	Baseline	0	-1.18	-0.38	0	0	0	0	8.61	7.90		
2012	Week #6	6	-1.19	-0.30	0	0	0	0	8.73	8.02		
2012	Week #12	12	-1.10	-0.23	0	0	0	0	8.84	8.13	N (not symp)	Cell count 200-499, moderate suppression
3001	Baseline	0	-0.58	-0.33	0	0	0	0	1.13	0.97	C (severely symp)	Cell count < 200, severe suppression
3001	Week #6	6	-0.43	-0.08	0	0	0	0	1.25	1.08		
3001	Week #12	12	-0.43	-0.13	0	0	0	0	1.36	1.20	C (severely symp)	Cell count < 200, severe suppression
3002	Baseline	0	-3.35	-3.35	1	1	1	0	8.65	8.65	B (moderately symp)	Cell count < 200, severe suppression
3002	Week #6	6	-3.50	-3.76	0	1	1	0	8.76	8.76		
3002	Week #12	12	-3.29	-3.28	0	1	1	0	8.90	8.90	B (moderately symp)	Cell count < 200, severe suppression
3003	Baseline	0	-0.70	-1.08	0	0	0	0	3.65	0.56	A (mildly symp)	Cell count 200-499, moderate suppression
3003	Week #6	6	-0.71	-1.20	0	0	0	0	3.76	0.67		
3003	Week #12	12	-0.41	-0.75	0	0	0	0	3.90	0.81	A (mildly symp)	Cell count 200-499, moderate suppression
3004	Baseline	0	-2.68	-1.78	0	1	0	0		6.66	B (moderately symp)	Cell count >= 500, no evidence of immune suppression
3004	Week #6	6	-2.38	-1.48	0	1	0	0		6.78		
3004	Week #12	12	-2.36	-1.49	0	1	0	0		6.89	B (moderately symp)	Cell count >= 500, no evidence of immune suppression
3005	Baseline	0	-2.02	-1.43	0	1	0	0	5.67	5.54	A (mildly symp)	Cell count 200-499, moderate suppression
3005	Week #6	6	-2.15	-1.61	0	1	0	0	5.78	5.66		
3005	Week #12	12	-2.11	-1.54	0	1	0	0	5.90	5.77	A (mildly symp)	Cell count 200-499, moderate suppression
3006	Baseline	0	-1.20	-0.02	1	0	0	0	8.67	8.67	B (moderately symp)	Cell count >= 500, no evidence of immune suppression
3006	Week #6	6	-1.12	0.03	1	0	0	0	8.79	8.79		
3006	Week #12	12	-1.15	-0.06	0	0	0	0	8.94	8.94	B (moderately symp)	Cell count >= 500, no evidence of immune suppression
3007	Baseline	0	-2.15	-1.25	0	1	0	0	11.68	5.68	C (severely symp)	Cell count >= 500, no evidence of immune suppression
3007	Week #6	6	-1.99	-1.22	0	0	0	0	11.80	5.80		
3007	Week #12	12	-2.06	-1.17	0	1	0	0	11.92	5.92	C (severely symp)	Cell count >= 500, no evidence of immune suppression
3008	Baseline	0	0.76	0.50	0	0	0	0	6.70	6.70	C (severely symp)	Cell count >= 500, no evidence of immune suppression

Botswana Vitamin D S

ID	Visit	Time wks	Weight-for-age Z Score	BMI-for-age Z Score	Height-for-age Z < -2.0 0=no, 1=yes	Weight-for-age Z < -2.0 0=no, 1=yes	BMI-for-age Z < -2.0 0=no, 1=yes	BMI category 0=<85th, 1=≥85th percentile	Years since HIV diagnosis	Years on HAART	CDC HIV Classification	CD4 count Category: worst
3008	Week #6	6	0.90	0.66	0	0	0	0	6.83	6.83		
3008	Week #12	12	0.94	0.75	0	0	0	0	6.94	6.94	C (severely symp)	Cell count >= 500, no evidence of immune suppression
3009	Baseline	0	-2.43	-0.87	1	1	0	0		6.79	A (mildly symp)	Cell count 200-499, moderate suppression
3009	Week #6	6	-2.70	-1.32	1	1	0	0		6.90		
3009	Week #12	12	-2.31	-0.70	1	1	0	0		7.03	A (mildly symp)	Cell count 200-499, moderate suppression
3010	Baseline	0	-6.22	-3.50	1	1	1	0	0.33	0.33	B (moderately symp)	Cell count < 200, severe suppression
3010	Week #6	6	-6.55	-3.80	1	1	1	0	0.44	0.44		
3010	Week #12	12	-6.13	-3.08	1	1	1	0	0.56	0.56	B (moderately symp)	Cell count < 200, severe suppression
3011	Baseline	0	-1.70	-3.17	0	0	1	0			C (severely symp)	Cell count 200-499, moderate suppression
3011	Week #6	6	-1.69	-3.16	0	0	1	0				
3011	Week #12	12	-1.36	-2.52	0	0	1	0			C (severely symp)	Cell count 200-499, moderate suppression
3012	Baseline	0	-0.91	0.12	0	0	0	0	6.73	5.73	C (severely symp)	Cell count 200-499, moderate suppression
3012	Week #6	6	-0.98	0.01	0	0	0	0	6.84	5.84		
3012	Week #12	12	-1.03	0.06	1	0	0	0	6.96	5.96	C (severely symp)	Cell count 200-499, moderate suppression
4001	Baseline	0							8.57	7.03	C (severely symp)	Cell count 200-499, moderate suppression
4001	Week #6	6							8.69	7.15		
4001	Week #12	12							8.80	7.26	C (severely symp)	Cell count 200-499, moderate suppression
4002	Baseline	0							9.66	3.51	A (mildly symp)	Cell count < 200, severe suppression
4002	Week #6	6							9.78	3.62		
4002	Week #12	12							9.89	3.74	A (mildly symp)	Cell count < 200, severe suppression
4003	Baseline	0							2.67	0.54	C (severely symp)	Cell count < 200, severe suppression
4003	Week #6	6							2.79	0.66		
4003	Week #12	12							2.91	0.79	C (severely symp)	Cell count < 200, severe suppression
4004	Baseline	0							3.69	2.69	A (mildly symp)	Cell count 200-499, moderate suppression
4004	Week #6	6							3.80	2.80		
4004	Week #12	12							3.92	2.92	A (mildly symp)	Cell count 200-499, moderate suppression
4005	Baseline	0							8.81	8.69	C (severely symp)	Cell count < 200, severe suppression
4005	Week #6	6							8.93	8.80		
4005	Week #12	12							9.04	8.92	C (severely symp)	Cell count < 200, severe suppression
4006	Baseline	0							7.70	3.52	B (moderately symp)	Cell count 200-499, moderate suppression
4006	Week #6	6							7.82	3.63		
4006	Week #12	12							7.93	3.75	B (moderately symp)	Cell count 200-499, moderate suppression
4007	Baseline	0							5.97	5.85	C (severely symp)	Cell count 200-499, moderate suppression
4007	Week #6	6							6.08	5.97		
4007	Week #12	12							6.20	6.08	C (severely symp)	Cell count 200-499, moderate suppression
4008	Baseline	0	-4.55	-4.89	0	1	1	0	9.76	4.76	C (severely symp)	Cell count < 200, severe suppression
4008	Week #6	6	-4.53	-4.79	0	1	1	0	9.88	4.88		
4008	Week #12	12	-4.29	-4.45	0	1	1	0	9.99	4.99	C (severely symp)	Cell count < 200, severe suppression
4009	Baseline	0	-2.56	-2.85	0	1	1	0		6.78	A (mildly symp)	Cell count 200-499, moderate suppression
4009	Week #6	6	-2.50	-2.79	0	1	1	0		6.89		
4009	Week #12	12	-2.65	-2.99	0	1	1	0		7.01	A (mildly symp)	Cell count 200-499, moderate suppression
4010	Baseline	0							1.49	0.66	C (severely symp)	Cell count 200-499, moderate suppression
4010	Week #6	6							1.62	0.79		
4010	Week #12	12							1.72	0.89	C (severely symp)	Cell count 200-499, moderate suppression
4011	Baseline	0	-1.07	-1.40	0	0	0	0	7.80	7.80	A (mildly symp)	Cell count >= 500, no evidence of immune suppression
4011	Week #6	6	-1.11	-1.51	0	0	0	0	7.92	7.92		
4011	Week #12	12	-1.06	-1.43	0	0	0	0	8.03	8.03	A (mildly symp)	Cell count >= 500, no evidence of immune suppression

Botswana Vitamin D S

ID	Visit	Time wks	Weight-for-age Z Score	BMI-for-age Z Score	Height-for-age Z < -2.0 0=no, 1=yes	Weight-for-age Z < -2.0 0=no, 1=yes	BMI-for-age Z < -2.0 0=no, 1=yes	BMI category 0=<85th, 1=≥85th percentile	Years since HIV diagnosis	Years on HAART	CDC HIV Classification	CD4 count Category: worst
4012	Baseline	0	-3.29	-2.19	1	1	1	0	3.80	2.67	C (severely symp)	Cell count < 200, severe suppression
4012	Week #6	6	-3.38	-2.43	1	1	1	0	3.92	2.79		
4012	Week #12	12	-3.64	-2.79	1	1	1	0	4.03	2.90	C (severely symp)	Cell count < 200, severe suppression
5001	Baseline	0							7.75	6.24	C (severely symp)	Cell count 200-499, moderate suppression
5001	Week #6	6							7.86	6.36		
5001	Week #12	12							7.98	6.47	C (severely symp)	Cell count 200-499, moderate suppression
5002	Baseline	0							4.94	4.83		
5002	Week #6	6							5.06	4.94		
5003	Baseline	0							7.07	6.99	C (severely symp)	Cell count < 200, severe suppression
5003	Week #6	6							7.19	7.10		
5003	Week #12	12							7.30	7.22	C (severely symp)	Cell count < 200, severe suppression
5004	Baseline	0							8.11	8.11	A (mildly symp)	Cell count < 200, severe suppression
5004	Week #6	6							8.23	8.23		
5004	Week #12	12							8.34	8.34	A (mildly symp)	Cell count < 200, severe suppression
5005	Baseline	0							8.47	7.22	C (severely symp)	Cell count < 200, severe suppression
5005	Week #6	6							8.58	7.33		
5005	Week #12	12							8.73	7.48	C (severely symp)	Cell count < 200, severe suppression
5006	Baseline	0							6.47	6.47	C (severely symp)	Cell count < 200, severe suppression
5006	Week #6	6							6.59	6.59		
5006	Week #12	12							6.70	6.70	C (severely symp)	Cell count < 200, severe suppression
5007	Baseline	0							6.52	6.52	C (severely symp)	Cell count 200-499, moderate suppression
5007	Week #6	6							6.63	6.63		
5007	Week #12	12							6.75	6.75	C (severely symp)	Cell count 200-499, moderate suppression
5008	Baseline	0							9.05	9.05	C (severely symp)	Cell count < 200, severe suppression
5008	Week #6	6							9.17	9.17		
5008	Week #12	12							9.28	9.28	C (severely symp)	Cell count < 200, severe suppression
5009	Baseline	0							6.32	6.25	C (severely symp)	Cell count < 200, severe suppression
5009	Week #6	6							6.44	6.37		
5009	Week #12	12							6.55	6.48	C (severely symp)	Cell count < 200, severe suppression
5010	Baseline	0							7.74	7.72	C (severely symp)	Cell count < 200, severe suppression
5010	Week #6	6							7.86	7.84		
5010	Week #12	12							7.97	7.95	C (severely symp)	Cell count < 200, severe suppression
5011	Baseline	0							5.54	5.24	C (severely symp)	Cell count < 200, severe suppression
5011	Week #6	6							5.68	5.39		
5011	Week #12	12							5.77	5.47	C (severely symp)	Cell count < 200, severe suppression
5012	Baseline	0							6.96	2.33	C (severely symp)	Cell count < 200, severe suppression
5012	Week #6	6							7.08	2.45		
5012	Week #12	12							7.18	2.55	C (severely symp)	Cell count < 200, severe suppression

Botswana Vitamin D S

ID	Visit	Time wks	CD4 count Category: present	Taking HAART	Taking NNRTI(s)	Taking NRTI(s)	Taking PIs	Taking Other HIV Medications	Taking Efavirenz 0=no, 1=yes	Taking Nevirapine 0=no, 1=yes	Taking Tenofovir 0=no, 1=yes	Taking Atripla 0=no, 1=yes	Taking Truvada 0=no, 1=yes
1001	Baseline	0	Cell count >= 500, no evidence of immune suppression	Yes	No	Yes	Yes	No	0	0	0	0	0
1001	Week #6	6		Yes	No	Yes	Yes	No	0	0	0	0	0
1001	Week #12	12	Cell count >= 500, no evidence of immune suppression	Yes	No	Yes	Yes	No	0	0	0	0	0
1002	Baseline	0	Cell count >= 500, no evidence of immune suppression	Yes	No	Yes	Yes	No	0	0	0	0	0
1002	Week #6	6		Yes	No	Yes	Yes	No	0	0	0	0	0
1002	Week #12	12	Cell count >= 500, no evidence of immune suppression	Yes	No	Yes	Yes	No	0	0	0	0	0
1003	Baseline	0	Cell count >= 500, no evidence of immune suppression	Yes	Yes	Yes	No	No	1	0	0	0	0
1003	Week #6	6		Yes	Yes	Yes	No	No	1	0	0	0	0
1003	Week #12	12	Cell count >= 500, no evidence of immune suppression	Yes	Yes	Yes	No	No	1	0	0	0	0
1004	Baseline	0	Cell count >= 500, no evidence of immune suppression	Yes	No	Yes	Yes	No	0	0	0	0	0
1004	Week #6	6		Yes	No	Yes	Yes	No	0	0	0	0	0
1004	Week #12	12	Cell count >= 500, no evidence of immune suppression	Yes	No	Yes	Yes	No	0	0	0	0	0
1005	Baseline	0	Cell count >= 500, no evidence of immune suppression	Yes	Yes	Yes	No	No	1	0	0	0	0
1005	Week #6	6		Yes	Yes	Yes	No	No	1	0	0	0	0
1005	Week #12	12	Cell count >= 500, no evidence of immune suppression	Yes	Yes	Yes	No	No	1	0	0	0	0
1006	Baseline	0	Cell count >= 500, no evidence of immune suppression	Yes	No	Yes	Yes	No	0	0	0	0	0
1006	Week #6	6		Yes	No	Yes	Yes	No	0	0	0	0	0
1006	Week #12	12	Cell count >= 500, no evidence of immune suppression	Yes	No	Yes	Yes	No	0	0	0	0	0
1007	Baseline	0		Yes	No	Yes	Yes	No	0	0	0	0	0
1007	Week #6	6		Yes	No	Yes	Yes	No	0	0	0	0	0
1007	Week #12	12		Yes	No	Yes	Yes	No	0	0	0	0	0
1008	Baseline	0	Cell count >= 500, no evidence of immune suppression	Yes	Yes	Yes	No	No	0	1	0	0	0
1008	Week #6	6		Yes	Yes	Yes	No	No	0	1	0	0	0
1008	Week #12	12	Cell count >= 500, no evidence of immune suppression	Yes	Yes	Yes	No	No	0	1	0	0	0
1009	Baseline	0	Cell count >= 500, no evidence of immune suppression	Yes	Yes	Yes	No	No	1	0	0	0	0
1009	Week #6	6		Yes	Yes	Yes	No	No	1	0	0	0	0
1009	Week #12	12	Cell count >= 500, no evidence of immune suppression	Yes	Yes	Yes	No	No	1	0	0	0	0
1010	Baseline	0	Cell count >= 500, no evidence of immune suppression	Yes	Yes	Yes	No	No	0	1	0	0	0
1010	Week #6	6		Yes	Yes	Yes	No	No	0	1	0	0	0
1010	Week #12	12	Cell count >= 500, no evidence of immune suppression	Yes	Yes	Yes	No	No	0	1	0	0	0
1011	Baseline	0	Cell count >= 500, no evidence of immune suppression	Yes	Yes	Yes	No	No	0	1	0	0	0
1011	Week #6	6		Yes	Yes	Yes	No	No	0	1	0	0	0
1011	Week #12	12	Cell count >= 500, no evidence of immune suppression	Yes	Yes	Yes	No	No	0	1	0	0	0
1012	Baseline	0	Cell count >= 500, no evidence of immune suppression	Yes	Yes	Yes	No	No	0	1	0	0	0
1012	Week #6	6		Yes	Yes	Yes	No	No	0	1	0	0	0
1012	Week #12	12	Cell count >= 500, no evidence of immune suppression	Yes	Yes	Yes	No	No	0	1	0	0	0
2001	Baseline	0		Yes	Yes	Yes	No	No	0	1	0	0	0
2001	Week #6	6		Yes	Yes	Yes	No	No	0	1	0	0	0
2001	Week #12	12		Yes	Yes	Yes	No	No	0	1	0	0	0
2002	Baseline	0		Yes	No	Yes	Yes	No	0	0	0	0	0
2002	Week #6	6		Yes	No	Yes	Yes	No	0	0	0	0	0
2002	Week #12	12		Yes	No	Yes	Yes	No	0	0	0	0	0
2003	Baseline	0	Cell count >= 500, no evidence of immune suppression	Yes	Yes	Yes	No	No	0	1	0	0	0
2003	Week #6	6		Yes	Yes	Yes	No	No	0	1	0	0	0
2003	Week #12	12	Cell count >= 500, no evidence of immune suppression	Yes	Yes	Yes	No	No	0	1	0	0	0
2004	Baseline	0	Cell count >= 500, no evidence of immune suppression	Yes	No	Yes	Yes	No	0	0	0	0	0
2004	Week #6	6		Yes	No	Yes	Yes	No	0	0	0	0	0

Botswana Vitamin D S

ID	Visit	Time wks	CD4 count Category: present	Taking HAART	Taking NNRTI(s)	Taking NRTI(s)	Taking PIs	Taking Other HIV Medications	Taking Efavirenz 0=no, 1=yes	Taking Nevirapine 0=no, 1=yes	Taking Tenofovir 0=no, 1=yes	Taking Atripla 0=no, 1=yes	Taking Truvada 0=no, 1=yes
2004	Week #12	12	Cell count >= 500, no evidence of immune suppression	Yes	No	Yes	Yes	No	0	0	0	0	0
2005	Baseline	0	Cell count >= 500, no evidence of immune suppression	Yes	Yes	Yes	No	No	1	0	0	0	0
2005	Week #6	6		Yes	Yes	Yes	No	No	1	0	0	0	0
2005	Week #12	12	Cell count >= 500, no evidence of immune suppression	Yes	Yes	Yes	No	No	1	0	0	0	0
2006	Baseline	0	Cell count >= 500, no evidence of immune suppression	Yes	Yes	Yes	No	No	0	1	0	0	0
2006	Week #6	6		Yes	Yes	Yes	No	No	0	1	0	0	0
2006	Week #12	12	Cell count >= 500, no evidence of immune suppression	Yes	Yes	Yes	No	No	0	1	0	0	0
2007	Baseline	0	Cell count 200-499, moderate suppression	Yes	Yes	Yes	No	No	1	0	0	0	0
2007	Week #6	6		Yes	Yes	Yes	No	No	1	0	0	0	0
2007	Week #12	12	Cell count 200-499, moderate suppression	Yes	Yes	Yes	No	No	1	0	0	0	0
2008	Baseline	0	Cell count >= 500, no evidence of immune suppression	Yes	Yes	Yes	No	No	0	1	0	0	0
2008	Week #6	6		Yes	Yes	Yes	No	No	0	1	0	0	0
2008	Week #12	12	Cell count >= 500, no evidence of immune suppression	Yes	Yes	Yes	No	No	0	1	0	0	0
2009	Baseline	0	Cell count < 200, severe suppression	Yes	Yes	Yes	No	No	1	0	0	0	0
2009	Week #6	6		Yes	Yes	Yes	No	No	1	0	0	0	0
2009	Week #12	12	Cell count < 200, severe suppression	Yes	Yes	Yes	No	No	1	0	0	0	0
2010	Baseline	0	Cell count >= 500, no evidence of immune suppression	Yes	Yes	Yes	No	No	1	0	0	0	0
2010	Week #6	6		Yes	Yes	Yes	No	No	1	0	0	0	0
2010	Week #12	12	Cell count >= 500, no evidence of immune suppression	Yes	Yes	Yes	No	No	1	0	0	0	0
2011	Baseline	0	Cell count >= 500, no evidence of immune suppression	Yes	Yes	Yes	No	No	1	0	0	0	0
2011	Week #6	6		Yes	Yes	Yes	No	No	1	0	0	0	0
2011	Week #12	12	Cell count >= 500, no evidence of immune suppression	Yes	Yes	Yes	No	No	1	0	0	0	0
2012	Baseline	0	Cell count >= 500, no evidence of immune suppression	Yes	Yes	Yes	No	No	0	1	0	0	0
2012	Week #6	6		Yes	Yes	Yes	No	No	0	1	0	0	0
2012	Week #12	12	Cell count >= 500, no evidence of immune suppression	Yes	Yes	Yes	No	No	0	1	0	0	0
3001	Baseline	0	Cell count 200-499, moderate suppression	Yes	Yes	Yes	No	No	0	1	0	0	0
3001	Week #6	6		Yes	Yes	Yes	No	No	0	1	0	0	0
3001	Week #12	12	Cell count 200-499, moderate suppression	Yes	Yes	Yes	No	No	0	1	0	0	0
3002	Baseline	0	Cell count >= 500, no evidence of immune suppression	Yes	Yes	Yes	No	No	0	1	0	0	0
3002	Week #6	6		Yes	Yes	Yes	No	No	0	1	0	0	0
3002	Week #12	12	Cell count >= 500, no evidence of immune suppression	Yes	Yes	Yes	No	No	0	1	0	0	0
3003	Baseline	0	Cell count 200-499, moderate suppression	Yes	Yes	Yes	No	No	0	1	0	0	0
3003	Week #6	6		Yes	Yes	Yes	No	No	0	1	0	0	0
3003	Week #12	12	Cell count 200-499, moderate suppression	Yes	Yes	Yes	No	No	0	1	0	0	0
3004	Baseline	0	Cell count >= 500, no evidence of immune suppression	Yes	No	Yes	Yes	No	0	0	0	0	0
3004	Week #6	6		Yes	No	Yes	Yes	No	0	0	0	0	0
3004	Week #12	12	Cell count >= 500, no evidence of immune suppression	Yes	No	Yes	Yes	No	0	0	0	0	0
3005	Baseline	0	Cell count 200-499, moderate suppression	Yes	Yes	Yes	No	No	0	1	0	0	0
3005	Week #6	6		Yes	Yes	Yes	No	No	0	1	0	0	0
3005	Week #12	12	Cell count 200-499, moderate suppression	Yes	Yes	Yes	No	No	0	1	0	0	0
3006	Baseline	0	Cell count >= 500, no evidence of immune suppression	Yes	Yes	Yes	No	No	0	1	0	0	0
3006	Week #6	6		Yes	Yes	Yes	No	No	0	1	0	0	0
3006	Week #12	12	Cell count >= 500, no evidence of immune suppression	Yes	Yes	Yes	No	No	0	1	0	0	0
3007	Baseline	0	Cell count >= 500, no evidence of immune suppression	Yes	No	Yes	Yes	No	0	0	0	0	0
3007	Week #6	6		Yes	No	Yes	Yes	No	0	0	0	0	0
3007	Week #12	12	Cell count >= 500, no evidence of immune suppression	Yes	No	Yes	Yes	No	0	0	0	0	0
3008	Baseline	0	Cell count >= 500, no evidence of immune suppression	Yes	No	Yes	Yes	No	0	0	0	0	0

Botswana Vitamin D S

ID	Visit	Time wks	CD4 count Category: present	Taking HAART	Taking NNRTI(s)	Taking NRTI(s)	Taking PIs	Taking Other HIV Medications	Taking Efavirenz 0=no, 1=yes	Taking Nevirapine 0=no, 1=yes	Taking Tenofovir 0=no, 1=yes	Taking Atripla 0=no, 1=yes	Taking Truvada 0=no, 1=yes
3008	Week #6	6		Yes	No	Yes	Yes	No	0	0	0	0	0
3008	Week #12	12	Cell count >= 500, no evidence of immune suppression	Yes	No	Yes	Yes	No	0	0	0	0	0
3009	Baseline	0	Cell count >= 500, no evidence of immune suppression	Yes	Yes	Yes	No	No	1	0	0	0	0
3009	Week #6	6		Yes	Yes	Yes	No	No	1	0	0	0	0
3009	Week #12	12	Cell count >= 500, no evidence of immune suppression	Yes	Yes	Yes	No	No	1	0	0	0	0
3010	Baseline	0	Cell count < 200, severe suppression	Yes	Yes	Yes	No	No	1	0	0	0	0
3010	Week #6	6		Yes	Yes	Yes	No	No	1	0	0	0	0
3010	Week #12	12	Cell count < 200, severe suppression	Yes	Yes	Yes	No	No	1	0	0	0	0
3011	Baseline	0	Cell count >= 500, no evidence of immune suppression	Yes	No	Yes	Yes	No	0	0	0	0	0
3011	Week #6	6		Yes	No	Yes	Yes	No	0	0	0	0	0
3011	Week #12	12	Cell count >= 500, no evidence of immune suppression	Yes	No	Yes	Yes	No	0	0	0	0	0
3012	Baseline	0	Cell count >= 500, no evidence of immune suppression	Yes	Yes	Yes	No	No	0	1	0	0	0
3012	Week #6	6		Yes	Yes	Yes	No	No	0	1	0	0	0
3012	Week #12	12	Cell count >= 500, no evidence of immune suppression	Yes	Yes	Yes	No	No	0	1	0	0	0
4001	Baseline	0	Cell count >= 500, no evidence of immune suppression	Yes	Yes	Yes	No	No	0	1	0	0	0
4001	Week #6	6		Yes	Yes	Yes	No	No	0	1	0	0	0
4001	Week #12	12	Cell count >= 500, no evidence of immune suppression	Yes	Yes	Yes	No	No	0	1	0	0	0
4002	Baseline	0	Cell count >= 500, no evidence of immune suppression	Yes	No	Yes	Yes	No	0	0	0	0	0
4002	Week #6	6		Yes	No	Yes	Yes	No	0	0	0	0	0
4002	Week #12	12	Cell count >= 500, no evidence of immune suppression	Yes	No	Yes	Yes	No	0	0	0	0	0
4003	Baseline	0	Cell count 200-499, moderate suppression	Yes	Yes	Yes	No	No	1	0	1	1	0
4003	Week #6	6		Yes	Yes	Yes	No	No	1	0	1	1	0
4003	Week #12	12	Cell count 200-499, moderate suppression	Yes	Yes	Yes	No	No	1	0	1	1	0
4004	Baseline	0	Cell count 200-499, moderate suppression	Yes	Yes	Yes	No	No	0	1	1	0	1
4004	Week #6	6		Yes	Yes	Yes	No	No	0	1	1	0	1
4004	Week #12	12	Cell count 200-499, moderate suppression	Yes	Yes	Yes	No	No	0	1	1	0	1
4005	Baseline	0	Cell count 200-499, moderate suppression	Yes	Yes	Yes	No	No	0	1	0	0	0
4005	Week #6	6		Yes	Yes	Yes	No	No	0	1	0	0	0
4005	Week #12	12	Cell count 200-499, moderate suppression	Yes	Yes	Yes	No	No	0	1	0	0	0
4006	Baseline	0	Cell count >= 500, no evidence of immune suppression	Yes	Yes	Yes	No	No	0	1	1	0	1
4006	Week #6	6		Yes	Yes	Yes	No	No	0	1	1	0	1
4006	Week #12	12	Cell count >= 500, no evidence of immune suppression	Yes	Yes	Yes	No	No	0	1	1	0	1
4007	Baseline	0	Cell count 200-499, moderate suppression	Yes	Yes	Yes	No	No	0	1	0	0	0
4007	Week #6	6		Yes	Yes	Yes	No	No	0	1	0	0	0
4007	Week #12	12	Cell count 200-499, moderate suppression	Yes	Yes	Yes	No	No	0	1	1	0	1
4008	Baseline	0	Cell count >= 500, no evidence of immune suppression	Yes	No	Yes	Yes	No	0	0	0	0	0
4008	Week #6	6		Yes	No	Yes	Yes	No	0	0	0	0	0
4008	Week #12	12	Cell count >= 500, no evidence of immune suppression	Yes	No	Yes	Yes	No	0	0	0	0	0
4009	Baseline	0	Cell count 200-499, moderate suppression	Yes	No	Yes	Yes	No	0	0	0	0	0
4009	Week #6	6		Yes	No	Yes	Yes	No	0	0	0	0	0
4009	Week #12	12	Cell count 200-499, moderate suppression	Yes	No	Yes	Yes	No	0	0	0	0	0
4010	Baseline	0	Cell count 200-499, moderate suppression	Yes	Yes	Yes	No	No	1	0	1	1	0
4010	Week #6	6		Yes	Yes	Yes	No	No	1	0	1	1	0
4010	Week #12	12	Cell count 200-499, moderate suppression	Yes	Yes	Yes	No	No	1	0	1	1	0
4011	Baseline	0	Cell count >= 500, no evidence of immune suppression	Yes	Yes	Yes	No	No	1	0	1	1	0
4011	Week #6	6		Yes	Yes	Yes	No	No	1	0	1	1	0
4011	Week #12	12	Cell count >= 500, no evidence of immune suppression	Yes	Yes	Yes	No	No	1	0	1	1	0

Botswana Vitamin D S

ID	Visit	Time wks	CD4 count Category: present	Taking HAART	Taking NNRTI(s)	Taking NRTI(s)	Taking PIs	Taking Other HIV Medications	Taking Efavirenz 0=no, 1=yes	Taking Nevirapine 0=no, 1=yes	Taking Tenofovir 0=no, 1=yes	Taking Atripla 0=no, 1=yes	Taking Truvada 0=no, 1=yes
4012	Baseline	0	Cell count < 200, severe suppression	Yes	Yes	Yes	No	No	1	0	1	1	0
4012	Week #6	6		Yes	Yes	Yes	No	No	1	0	1	1	0
4012	Week #12	12	Cell count < 200, severe suppression	Yes	Yes	Yes	No	No	1	0	1	1	0
5001	Baseline	0	Cell count >= 500, no evidence of immune suppression	Yes	Yes	Yes	No	No	0	1	1	0	1
5001	Week #6	6		Yes	Yes	Yes	No	No	0	1	1	0	1
5001	Week #12	12	Cell count >= 500, no evidence of immune suppression	Yes	Yes	Yes	No	No	0	1	1	0	1
5002	Baseline	0		Yes	Yes	Yes	No	No	0	1	0	0	0
5002	Week #6	6		Yes	Yes	Yes	No	No	0	1	0	0	0
5003	Baseline	0	Cell count >= 500, no evidence of immune suppression	Yes	Yes	Yes	No	No	0	1	0	0	0
5003	Week #6	6		Yes	Yes	Yes	No	No	0	1	0	0	0
5003	Week #12	12	Cell count >= 500, no evidence of immune suppression	Yes	Yes	Yes	No	No	0	1	0	0	0
5004	Baseline	0	Cell count >= 500, no evidence of immune suppression	Yes	No	Yes	Yes	No	0	0	0	0	0
5004	Week #6	6		Yes	No	Yes	Yes	No	0	0	0	0	0
5004	Week #12	12	Cell count >= 500, no evidence of immune suppression	Yes	Yes	Yes	No	No	1	0	0	0	0
5005	Baseline	0	Cell count >= 500, no evidence of immune suppression	Yes	Yes	Yes	No	No	0	1	0	0	0
5005	Week #6	6		Yes	Yes	Yes	No	No	0	1	0	0	0
5005	Week #12	12	Cell count >= 500, no evidence of immune suppression	Yes	Yes	Yes	No	No	0	1	0	0	0
5006	Baseline	0	Cell count 200-499, moderate suppression	Yes	Yes	Yes	No	No	1	0	0	0	0
5006	Week #6	6		Yes	Yes	Yes	No	No	1	0	0	0	0
5006	Week #12	12	Cell count 200-499, moderate suppression	Yes	Yes	Yes	No	No	1	0	0	0	0
5007	Baseline	0	Cell count 200-499, moderate suppression	Yes	Yes	Yes	No	No	0	1	0	0	0
5007	Week #6	6		Yes	Yes	Yes	No	No	0	1	0	0	0
5007	Week #12	12	Cell count 200-499, moderate suppression	Yes	Yes	Yes	No	No	0	1	0	0	0
5008	Baseline	0	Cell count >= 500, no evidence of immune suppression	Yes	Yes	Yes	No	No	1	0	1	1	0
5008	Week #6	6		Yes	Yes	Yes	No	No	1	0	1	1	0
5008	Week #12	12	Cell count >= 500, no evidence of immune suppression	Yes	Yes	Yes	No	No	1	0	1	1	0
5009	Baseline	0	Cell count >= 500, no evidence of immune suppression	Yes	Yes	Yes	No	No	1	0	0	0	0
5009	Week #6	6		Yes	Yes	Yes	No	No	1	0	0	0	0
5009	Week #12	12	Cell count >= 500, no evidence of immune suppression	Yes	Yes	Yes	No	No	1	0	0	0	0
5010	Baseline	0	Cell count 200-499, moderate suppression	Yes	Yes	Yes	No	No	1	0	0	0	0
5010	Week #6	6		Yes	Yes	Yes	No	No	1	0	0	0	0
5010	Week #12	12	Cell count 200-499, moderate suppression	Yes	Yes	Yes	No	No	1	0	0	0	0
5011	Baseline	0	Cell count 200-499, moderate suppression	Yes	Yes	Yes	No	No	1	0	0	0	0
5011	Week #6	6		Yes	Yes	Yes	No	No	1	0	0	0	0
5011	Week #12	12	Cell count 200-499, moderate suppression	Yes	Yes	Yes	No	No	1	0	0	0	0
5012	Baseline	0	Cell count 200-499, moderate suppression	Yes	Yes	Yes	No	No	1	0	0	0	0
5012	Week #6	6		Yes	Yes	Yes	No	No	1	0	0	0	0
5012	Week #12	12	Cell count 200-499, moderate suppression	Yes	Yes	Yes	No	No	1	0	0	0	0
